# ABI4 Regulates Primary Seed Dormancy by Regulating the Biogenesis of Abscisic Acid and Gibberellins in Arabidopsis

**DOI:** 10.1371/journal.pgen.1003577

**Published:** 2013-06-20

**Authors:** Kai Shu, Huawei Zhang, Shengfu Wang, Mingluan Chen, Yaorong Wu, Sanyuan Tang, Chunyan Liu, Yuqi Feng, Xiaofeng Cao, Qi Xie

**Affiliations:** 1 State Key Laboratory of Plant Genomics, National Center for Plant Gene Research, Institute of Genetics and Developmental Biology, Chinese Academy of Sciences, Beijing, P. R. China; 2 University of Chinese Academy of Sciences, Beijing, P. R. China; 3 Key Laboratory of Analytical Chemistry for Biology and Medicine (Ministry of Education), Department of Chemistry, Wuhan University, Wuhan, P. R. China; National University of Singapore and Temasek Life Sciences Laboratory, Singapore

## Abstract

Seed dormancy is an important economic trait for agricultural production. Abscisic acid (ABA) and Gibberellins (GA) are the primary factors that regulate the transition from dormancy to germination, and they regulate this process antagonistically. The detailed regulatory mechanism involving crosstalk between ABA and GA, which underlies seed dormancy, requires further elucidation. Here, we report that ABI4 positively regulates primary seed dormancy, while negatively regulating cotyledon greening, by mediating the biogenesis of ABA and GA. Seeds of the Arabidopsis *abi4* mutant that were subjected to short-term storage (one or two weeks) germinated significantly more quickly than Wild-Type (WT), and *abi4* cotyledons greened markedly more quickly than WT, while the rates of germination and greening were comparable when the seeds were subjected to longer-term storage (six months). The ABA content of dry *abi4* seeds was remarkably lower than that of WT, but the amounts were comparable after stratification. Consistently, the GA level of *abi4* seeds was increased compared to WT. Further analysis showed that *abi4* was resistant to treatment with paclobutrazol (PAC), a GA biosynthesis inhibitor, during germination, while *OE-ABI4* was sensitive to PAC, and exogenous GA rescued the delayed germination phenotype of *OE-ABI4*. Analysis by qRT-PCR showed that the expression of genes involved in ABA and GA metabolism in dry and germinating seeds corresponded to hormonal measurements. Moreover, chromatin immunoprecipitation qPCR (ChIP-qPCR) and transient expression analysis showed that ABI4 repressed *CYP707A1* and *CYP707A2* expression by directly binding to those promoters, and the ABI4 binding elements are essential for this repression. Accordingly, further genetic analysis showed that *abi4* recovered the delayed germination phenotype of *cyp707a1* and *cyp707a2* and further, rescued the non-germinating phenotype of *ga1-t*. Taken together, this study suggests that ABI4 is a key factor that regulates primary seed dormancy by mediating the balance between ABA and GA biogenesis.

## Author Summary

Seed dormancy prevents or delays germination in maturated seeds. The optimal level of seed dormancy is a valuable trait for agricultural production and post-harvest management. High ABA and low GA content in seeds promote seed dormancy. However, the precise molecular mechanisms controlling seed dormancy and germination remain unclear. We found that ABI4, the key transcription factor in the ABA signaling pathway, indeed controls primary seed dormancy. This result contradicts the previous conclusion that ABI4 is not involved in the control of seed dormancy. Several lines of evidence support our conclusion. For example, detailed physiological analysis of the germination of *abi4* seeds that were harvested immediately and stored for various periods of time and subjected to various treatments allowed us to conclude that ABI4 negatively regulates primary seed dormancy. The molecular mechanism responsible for this control is as follows: ABI4 directly or indirectly regulates the key genes of the ABA and GA biogenesis pathways, which then regulates the ABA and GA contents in seeds. Importantly, further genetic interactions between *CYP707A1*, *CYP707A2*, *GA1*, and *ABI4* also support our conclusion.

## Introduction

Seed dormancy prevents or delays the germination of maturated seeds even when conditions are favorable for germination [1]–[3]. Seed dormancy is an important trait for diverse, important crop species including rapeseed, wheat, corn and rice, because seed dormancy inhibits pre-harvest spouting or vivipary [4]. Vivipary usually causes great economic loss to cereal production, including losses in seed quantity and quality, especially in humid regions worldwide [5], [6]. On the other hand, deep seed dormancy is problematic, especially in the horticultural and forest industries, and chemical treatments may be required to promote germination [7]. Thus, the optimal level of seed dormancy is a valuable trait for agricultural production. Therefore, it is essential to understand the precise molecular mechanisms that control seed dormancy and germination.

Diverse endogenous and environmental factors including phytohormones, nutrients, temperature and light affect seed dormancy through different pathways [3], [8]. Extensive studies have shown that abscisic acid (ABA) and gibberellin acid (GA) are the primary endogenous factors that regulate the transition from dormancy to germination, and they regulate this process antagonistically [1], [2], [9]–[11]. ABA is essential for the induction and maintenance of seed dormancy, while GA is required for the release of dormancy and for the initiation of seed germination [9], [10]. In line with this conclusion, some ABA-deficient mutants such as *nced6*, *nced3*, *nced5*, *nced9*, *aba2* and *aao3* are better able to germinate than WT seeds [9], [12]. In support of these observations, the overexpression of the ABA biosynthesis gene *ABA2* enhances ABA accumulation and maintains deep seed dormancy [13]. Further, overexpression of other ABA biosynthesis genes, *NCED6* and *NCED9*, even inhibits precocious germination of developing seeds due to increased ABA biogenesis [14]. By contrast, some ABA metabolic pathway mutants, such as *cyp707a1*, *cyp707a2* and *cyp707a3*, accumulate higher ABA levels than WT and subsequently exhibit hyperdormancy in seeds [15]–[17]. In addition to ABA content, ABA signaling also positively regulates seed dormancy [2], [3]. Although ABI1 and ABI2 are negative regulators in the ABA signaling pathway, the *abi1-1* and *abi2-1* mutants show the reduced dormancy levels [18]. This phenotype results from dominant-negative mutations and therefore, these two PP2Cs (Protein Phosphatases type 2C) are unable to bind to ABA receptors [19], [20]. In addition, the *abi3* mutant also shows reduced seed dormancy levels [21]. Furthermore, the allelic mutant *abi3-3* even rescues the non-germinating phenotype of *ga1* in the absence of exogenous GA treatment, indicating that ABI3 is a negative regulator of GA biosynthesis [22]. Although a previous study has concluded that *abi5* does not reduce seed dormancy [23], other studies have shown that this gene negatively regulates seed germination [24], [25].

In contrast to ABA, GA negatively regulates seed dormancy [2], [3]. Mutants severely defective in GA biosynthesis such as *ga1* show deep seed dormancy and fail to germinate in the absence of exogenous GA [26]. On the other hand, mutants defective in GA 2-oxidases (GA2ox), which deactivate bioactive GA, exhibit reduced seed dormancy and germinate normally, even in the dark [27]. Mutations in two negative regulators in the GA signal transduction pathway, *rgl2* (*RGA-LIKE2*) and *spy* (*SPINDLY*), rescue the non-germination phenotype of *ga1-3* in the absence of exogenous GA [28], [29]. Combined with the conclusion that ABA and GA regulate seed dormancy antagonistically [3], the ability to synthesize GA is enhanced in the *aba2* mutant, indicating that ABA is involved in the suppression of GA biogenesis in both developing and imbibed seeds [9]. These pioneering studies demonstrated that ABA and GA biogenesis and signaling play key roles in the control of seed dormancy and germination. However, the detailed molecular mechanism by which the crosstalk between ABA and GA at the hormone biogenesis level regulates seed dormancy is largely unknown.

ABI4 encodes an AP2/ERF transcription factor, which is an enhancer in the ABA signal transduction pathway that functions especially during seed development and germination [23], [30], [31]. Furthermore, *ABI4* is also involved in other aspects of plant development including lipid mobilization from the embryo [32], glucose responses [33], [34], salt responses [35] and the mitochondrial and chloroplast-nucleus retrograde signaling pathways [36]–[38]. Most recently, ABI4 was found to regulate ABA and cytokinin inhibition of lateral root development by reducing polar auxin transport [39], as well as ABA- and jasmonate-dependent signaling pathway crosstalk [40] and the nitrogen deficiency stress response [41]. Except in young seedlings, the *ABI4* transcript level is relatively low through most stages of vegetative growth but high in both developing and imbibed seeds [31]. The abundance of ABI4 protein is partially regulated by the 26S-proteasomal pathway [42]. These excellent studies demonstrate that ABI4 is a versatile factor, which functions in diverse signaling pathways and is tightly regulated at the post-transcriptional level. However, the role of ABI4 in crosstalk between ABA and GA has not yet been elucidated.

As described above, many mutants in which the ABA signal is attenuated, such as *abi1-1*, *abi2-1* and *abi3*, exhibit the reduced seed dormancy phenotype [18], [21]. Although this protein is a positive regulator of the ABA signaling pathway, however, previous studies have concluded that ABI4 has no effect on seed dormancy [23], and this opinion has been accepted in the field [3], [43]. Recently, two studies have demonstrated that a mutation in a double-repeat AP2 domain transcription factor, *CHOTTO1*, results in reduced primary seed dormancy, and, interestingly, *ABI4* likely acts upstream of *CHOTTO1* in the genetic pathway [10], [44]. On the other hand, the *ABI4* transcript level is relatively low at almost all growth stages except during seed maturity and germination [31]. These studies inspired us to reconfirm the effects of ABI4 on primary seed dormancy as well as postgerminative growth.

Here, we show that *abi4* mutant seeds indeed exhibited reduced primary seed dormancy and increased cotyledon greening. The differences in germination rates and cotyledon greening between *abi4* and WT decreased moderately after stratification. After-ripening treatment caused the rates of germination and cotyledon greening to be comparable between *abi4* and WT. In line with these results, the ABA content in *abi4* dry seeds was significantly lower than that in WT, but the ABA levels were comparative after stratification treatment. Consistently, the GA level in *abi4* seeds was upregulated compared to WT. Further analysis showed that *abi4* was resistant to exogenous paclobutrazol (PAC), a GA biosynthesis inhibitor, while *OE-ABI4* was sensitive to PAC during germination, and exogenous GA rescued the delayed germination phenotype of *OE-ABI4*. The qRT-PCR assay also showed that the transcript levels of some GA biosynthesis and ABA inactivation genes were upregulated in germinating *abi4* seeds, while some GA inactivation and ABA biosynthesis genes were downregulated. ChIP-qPCR and transient expression assays showed that ABI4 indeed inhibits *CYP707A1* and *CYP707A2* transcription by directly binding to these promoters, and the CCAC *cis*-elements are essential for this repression. Further genetic analysis showed that *abi4* restored the delayed germination phenotype of *cyp707a1* and *cyp707a2*, and importantly, mutation in *ABI4* also rescued the non-germinating phenotype of *ga1-t* even in the absence of exogenous GA treatment, reconfirming that ABI4 is a negative regulator of GA biogenesis and a positive regulator of ABA biosynthesis during seed germination. Taken together, this study demonstrates that ABI4 plays pivotal and complex roles in fine-tuning the ABA/GA balance to control primary seed dormancy.

## Results

### Mutation in *ABI4* Reduces Primary Seed Dormancy

Occasionally we found that the *abi4-1* seeds germinated more quickly than WT seeds when the siliques fell onto the surface of the soil. Thus, we decided to investigate the effect of the *ABI4* gene on seed dormancy. The *abi4-1* (hereafter referred to as *abi4*) mutant was obtained from ABRC (Arabidopsis Biological Resource Center at the Ohio State University; stock number CS8104). This mutant contains a point mutation in the open reading frame in which the 469^th^ base G is deleted, resulting in a frame shift at codon 157 and producing a protein containing the predicted DNA binding and dimerization domains but lacking the presumed activation domain [31].

**Figure 1:**
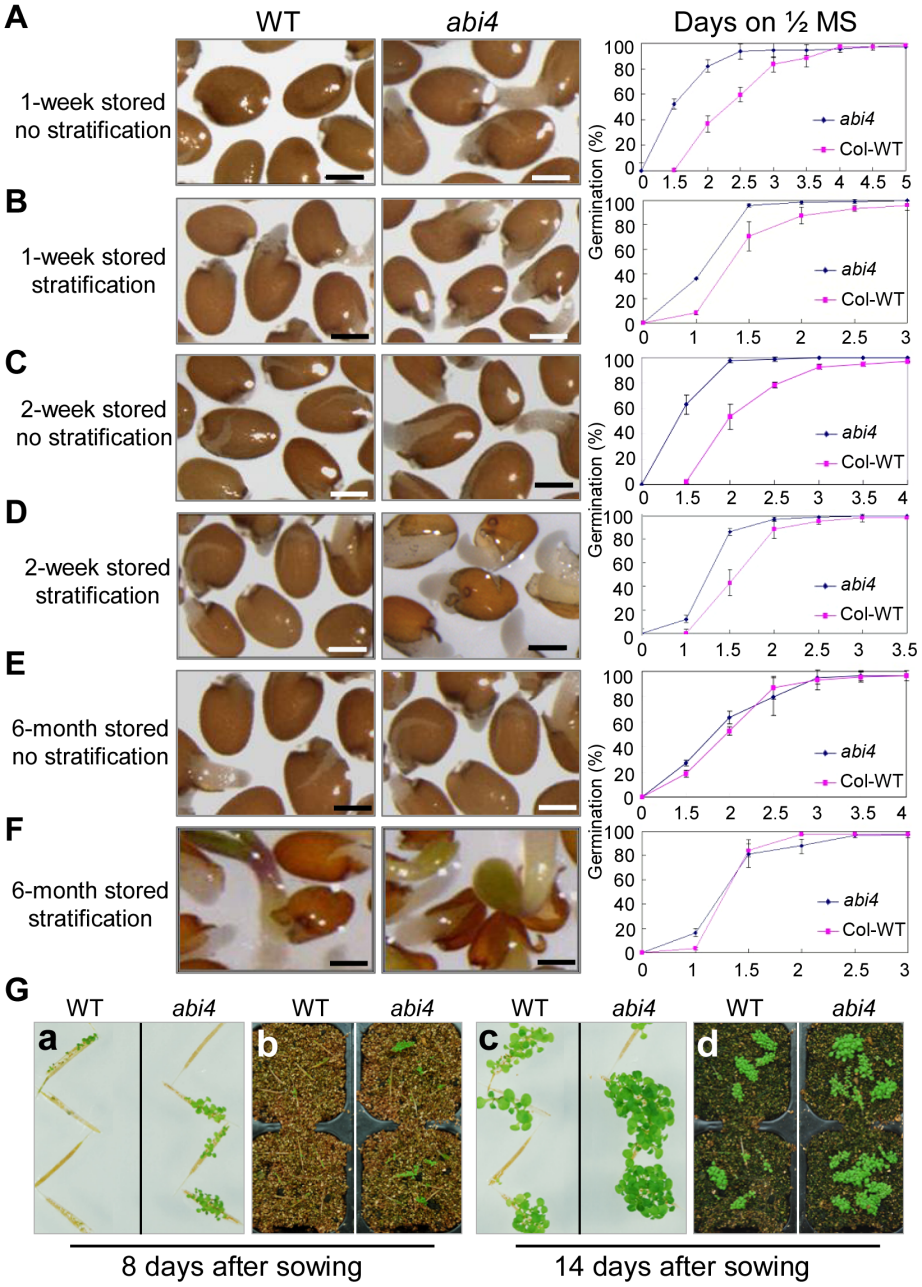
Decreased primary seed dormancy and vivipary phenotype of *abi4*. (A)–(F) Germination of WT and *abi4* seeds on 1/2 MS medium with or without stratification treatment. Seeds were stored for 1, 2 weeks or 6 months after harvest and subjected to analysis. Quantitative analysis of germination rates are shown in the right panels (n≥45). One representative image per genotype (1.5 days after sowing) is shown (left panels). Bar = 0.25 mm. Percentages are the average of three repeats ± standard error. (G) Representative images of vivipary phenotype of *abi4* on 1/2 MS medium (a, c) or on soil (b, d) are shown. Immature long-green siliques were collected from plants with various genotypes plants grown under the identical growth conditions.

To investigate the effect of *abi4* on seed dormancy, the germination of the *abi4* mutant and WT seeds was scored. Using seeds subjected to one week of dry storage, the germination rate of *abi4* seeds was clearly shown to be significantly higher than that of WT without 4°C stratification treatment (Figure 1A). At 1.5 days after sowing, the endosperms of most *abi4* seeds were ruptured, and radicles emerged from some seeds, while the testas of WT seeds had not even ruptured at this time (Figure 1A). The germination rate was nearly 80% for *abi4* seeds at day 2; however, the germination rate for WT seeds was less than 40% at this time point (Figure 1A). Consistent with the reduced dormancy level, the *abi4* mutant also exhibited markedly faster cotyledon greening than the WT (Figure S1A). In addition, it is noteworthy that at 4.5 days after sowing, the germination rates of *abi4* and WT reached 100% (Figure 1A). Taken together, the results of this time-course experiment show that the *abi4* mutant indeed exhibits reduced seed dormancy.

Previous studies have shown that stratification treatment reduces primary seed dormancy and thus promotes seed germination [10], [45]. Therefore, we also investigated the effect of stratification on primary seed dormancy in *abi4*. When seeds subjected to one week of dry storage were stratified for 3 days, the differences in the rates of germination and cotyledon greening between *abi4* and WT were moderately reduced (Figures 1B, S1B), compared with the larger difference shown in Figures 1A and S1A. However, the percentage of germination and cotyledon greening of *abi4* was still higher than that of WT, and the growth rate of the radicle of *abi4* was significantly faster than that of WT (Figures 1B, S1B). Next, when we examined seeds subjected to two weeks of dry-storage, the similar trends were detected (Figures 1C, S1C), and the differences between *abi4* and WT decreased moderately with stratification treatment (Figures 1D, S1D). Indeed, the *abi4* seeds subjected to short-term storage germinated more quickly than WT, especially without stratification treatment (Figure 1A, 1B, 1C and 1D). Subsequently, the effect of after-ripening on primary seed dormancy was investigated. The faster germination phenotype of *abi4* seeds was abolished when the seeds were fully after-ripened, either with or without stratification treatment (6-month dry-storage; Figure 1E, 1F). Consistent with these results, we also did not detect differences in cotyledon greening rates between *abi4* and WT when we employed fully after-ripened seeds (Figure S1E, S1F). Altogether, these results suggest that *abi4* reduces primary seed dormancy.

A reduced primary seed dormancy level usually results in preharvest sprouting or vivipary in cereals, especially if moist conditions are encountered [46].Therefore, we tested whether the *abi4* mutant exhibits vivipary in developing seeds using a protocol employed in a previous study [14]. The results show that *abi4* seeds in developing siliques indeed germinated more quickly than WT both on 1/2 MS medium and on soil (Figure 1G). In particular, at 8 days after sowing, only a few seeds in WT siliques germinated, while young *abi4* seedlings were already established (Figure 1Ga, 1Gb). At 14 days after sowing, most of the *abi4* and WT siliques produced seeds that germinated, and the cotyledons greened (Figure 1Gc, 1Gd), indicating that the seed vigor in these developing siliques was normal. Therefore, we reasoned that the difference in germination rate between *abi4* and WT siliques resulted from different seed dormancy levels.

To further confirm that the reduced primary dormancy level phenotype of *abi4* resulted from a mutation in the *ABI4* locus, we obtained another T-DNA insertion mutant in this locus from ABRC (the stock name is SALK_080095, hereafter referred to as *abi4-t*). A previous study showed that this line is a knockout mutant [47]. Similar to *abi4*, the decreased seed dormancy level, early germination phenotype of *abi4-t* was also observed when we analyzed seeds subjected to one week of dry storage (Figure S2A), and the percentage of cotyledon greening of *abi4-t* was also significantly higher than that of WT seeds (Figure S2B). On the other hand, the faster germination phenotype of *abi4-t* seeds (compared with WT) was abolished when fully after-ripened seeds were employed (data not shown). The similar phenotype of the two allele mutants further proved that a mutation in the *ABI4* locus is indeed responsible for the reduced primary seed dormancy phenotype.

To demonstrate the reproductivity of our experiment, we employed several mutants with seed dormancy phenotype in ABA pathway as controls. Previous study demonstrated that *snrk2.2/snrk2.3* obviously reduced seed dormancy level compared to WT [48]. This phenotype is resulted from the impaired ABA signaling in this double mutant [48]. In our growth condition, the reduced seed dormancy phenotype of *snrk2.2/snrk2.3* was repeated perfectly (Figure S3A). Notably, the decreased seed dormancy phenotype of *snrk2.2/snrk2.3* was stronger than that of *abi4* (Figure S3A). Furthermore, the reduced seed dormancy phenotype of *abi1-1* and *abi2-1* compared to Ler seeds was also detected in the same condition (Figure S3B), which was consistent with our current knowledge [18]. These results demonstrated that the present experimental condition is eligible and reliable.

### ABA Content Is Downregulated in *abi4* Seeds

**Figure 2:**
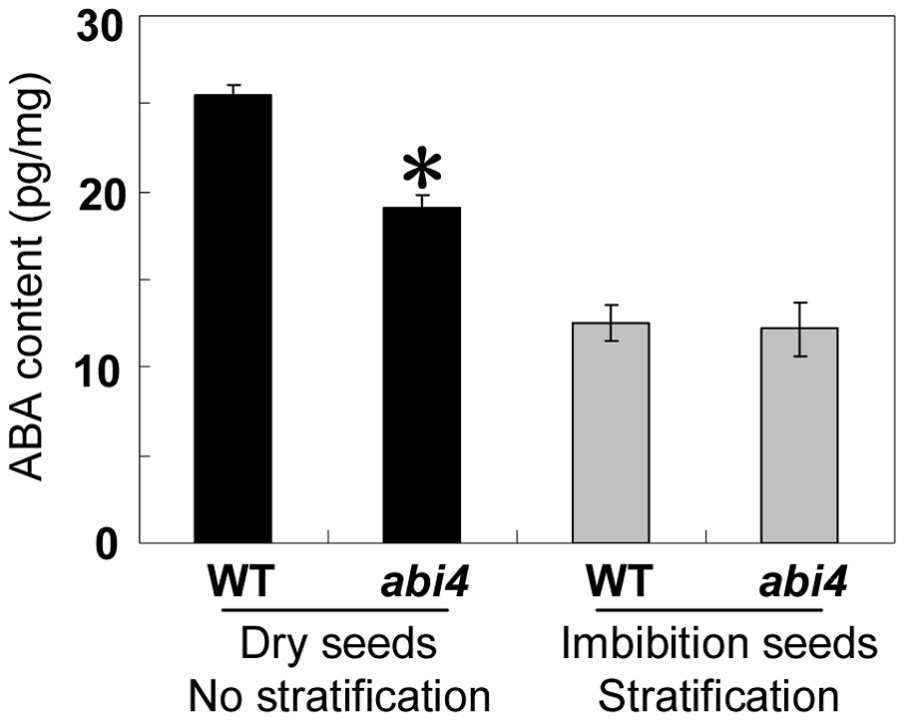
ABA quantification in *abi4* and WT dry and imbibed seeds. ABA contents were determined in dry and imbibed WT and *abi4* mutant seeds. Two-week stored seeds were used for analysis. The * stands for significant level of 0.05.

As described above, *abi4* seeds subjected to short-term storage exhibited significantly reduced dormancy compared with WT seeds, but the difference in germination rate between *abi4* and WT was decreased moderately or even abolished after stratification or longer period of storage (Figure 1). Since ABA positively regulates seed dormancy [1], and stratification and after-ripening treatment reduce the ABA content [10], we next examined endogenous ABA levels in dry and stratified *abi4* seeds using a liquid chromatography–tandem mass spectrometry system. We chose seeds subjected to two weeks of dry storage for this experiment. As expected, the result showed that the ABA content in *abi4* was significantly lower than that in WT (Figure 2) when no stratified seeds were analyzed, suggesting that ABI4 positively regulates ABA biogenesis. On the other hand, stratification treatment impairs ABA biosynthesis [49]. Accordingly, after a 3-day stratification treatment, both WT and *abi4* mutant seeds contained lower ABA levels, and importantly, the ABA levels were comparable between WT and *abi4* (Figure 2). The trend of ABA level in dry or stratified *abi4* seeds is similar to that of *CHOTTO1*, a positive regulator of primary seed dormancy that may acts downstream of *ABI4* in a genetic pathway [10], [44]. The hormonal measurements described above revealed that the decreased ABA level in *abi4* seeds is at least partially responsible for the reduced primary seed dormancy phenotype of this mutant (Figure 2).

### *OE-ABI4* Seeds Were Sensitive to PAC during Germination, while *abi4* Was Resistant

**Figure 3:**
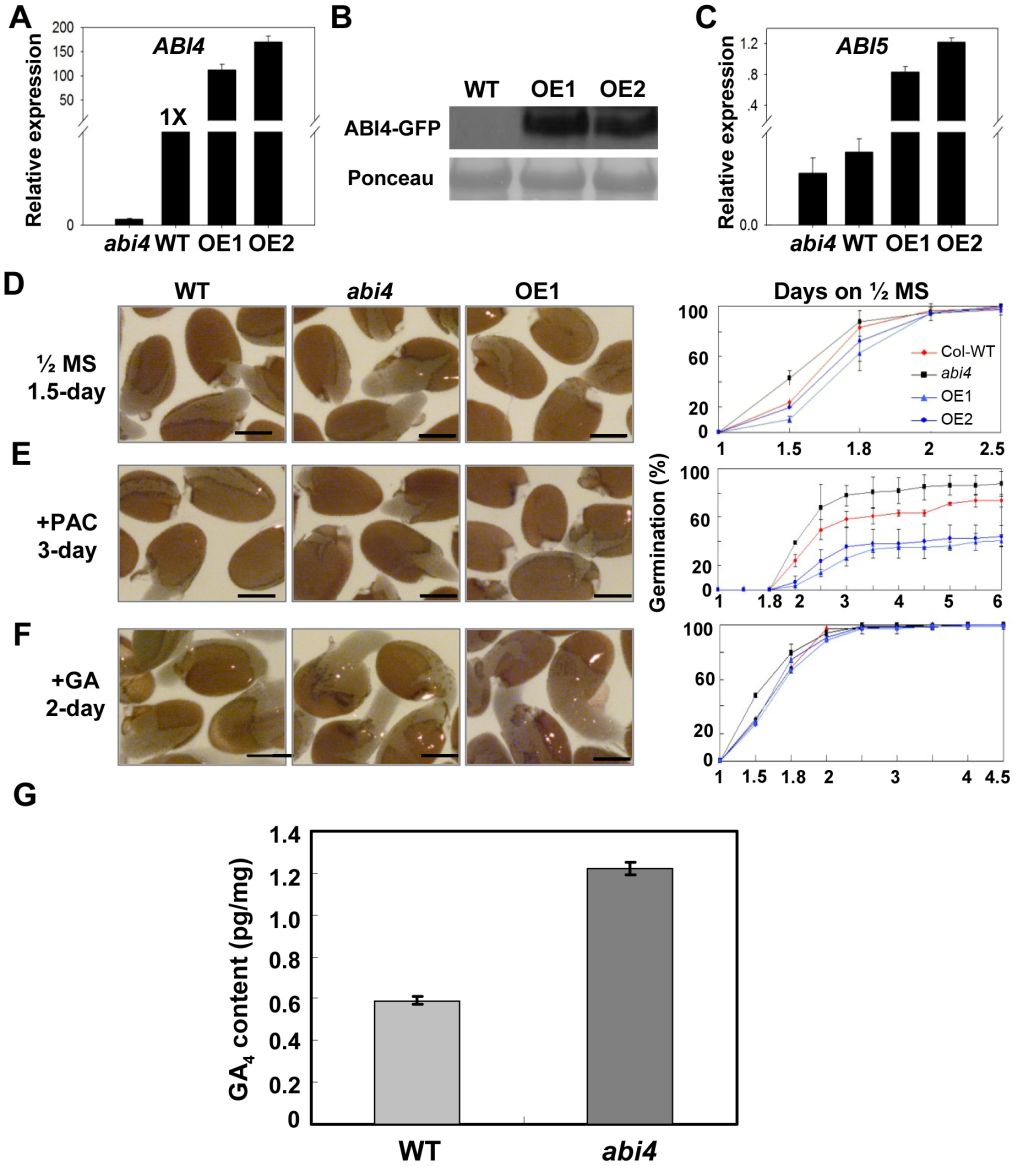
GA biogenesis is impaired in seeds of *abi4* mutant. (A) Two independent homozygous *OE-ABI4* lines were identified through qRT-PCR. (B) Western blot confirmed the two *OE-ABI4* transgenic lines. (C) *ABI5* expression analysis in *abi4*, WT and two *OE-ABI4* transgenic lines. (D)–(F) Germination analysis of WT, *abi4*, OE-1 and OE-2 seeds on 1/2 MS medium (D) 1/2 MS medium supplemented with 15 µM PAC. (E) 1/2 MS medium supplemented with 0.5 µM GA (F). Quantitative analysis of germination rates are shown in the right panels (n≥45). One representative image (time points indicated in figures) per genotype is shown (left panels). Bar = 0.25 mm. Percentages are average of three repeats ± standard error. (G) Endogenous GA_4_ levels in *abi4* and WT seeds were determined. Two-week stored seeds were used for analysis. Percentages are average of three repeats ± standard error.

To further investigate the precise mechanism by which ABI4 regulates primary seed dormancy, *ABI4*-overexpressing plants were generated. The coding region of *ABI4* was introduced into the vector *pCanG-HA-GFP* under the control of the CaMV (Cauliflower mosaic virus) 35S promoter and transformed into WT Arabidopsis. Several independent T3 homozygous lines were identified through qRT-PCR and western blot assays, and two of them were shown (Figure 3A, 3B). Because ABI4 directly promotes *ABI5* transcription [50], we examined the *ABI5* expression levels in those transgenic lines. The qRT-PCR assay showed that *ABI5* transcription was indeed upregulated in these *ABI4* overexpressing lines (Figure 3C). Thus, we reasoned that these two overexpressing lines are functional and they were employed in further analysis.

Because both the *abi4* and *abi4-t* mutants showed the reduced primary seed dormancy phenotype (Figures 1, S2), we first tested the seed dormancy level of *OE-ABI4* seeds subjected to two-week dry-storage on normal 1/2 MS medium. The results showed that the two independent lines germinated slowly than WT (Figure 3D), and accordingly, the cotyledon greening rates of these two lines were also moderately lower than that of WT (Figure S4A). These results indicate that the seed dormancy level in *OE-ABI4* seeds was higher than that of WT, which is in contrast to the phenotype of the both of *abi4* mutants.

Our results show that ABI4 positively regulates ABA biogenesis (Figure 2), and a previous study demonstrated that ABA is involved in the suppression of GA biosynthesis in imbibed seeds [9]. Thus, we speculated that the GA level in *abi4* was higher than that in WT. To confirm this speculation, we analyzed the responsiveness of *abi4* mutant and *OE-ABI4* seeds to GA and PAC treatment. Our results showed that *OE-ABI4* seeds were sensitive to PAC during germination (Figure 3E) and cotyledon greening (Figure S4B), while *abi4* was resistant (Figure 3E, S4B). However, the rates of germination and cotyledon greening among *abi4*, WT and *OE-ABI4* were comparable when we used medium supplemented with exogenous GA (Figure 3F, S4C). The increased resistance of *abi4* to the GA biosynthesis inhibitor suggests that this mutant contains higher levels of active GA or possesses stronger GA signaling than the WT [51]. Combined with the fact that exogenous GA can rescue the delayed germination and cotyledon greening phenotypes of *OE-ABI4*, we proposed that ABI4 attenuates GA biosynthesis to positively regulate seed dormancy.

### Active GA_4_ Content Is Upregulated in *abi4* Seeds

The responsiveness analysis of *abi4* and *OE-ABI4* seeds to GA and PAC treatments suggested that ABI4 negatively regulates GA biogenesis (Figure 3E, 3F). Furthermore, because that ABA is involved in the suppression of GA biosynthesis during seed germination [9], and the ABA measurements between *abi4* and WT seeds also supported this speculation (Figure 2). Then, we examined the endogenous GA content in *abi4* and WT seeds. The result showed that the active GA_4_ level in *abi4* dry seeds was significantly higher than that in WT (Figure 3G), suggesting that ABI4 indeed regulates GA biosynthesis negatively. Combined with the ABA quantification result, the endogenous hormone measurements demonstrated that the decreased ABA level and the increased GA content in *abi4* seeds are responsible for the reduced primary seed dormancy of *abi4*.

### Gene Expression Analysis in Dry and Imbibed Seeds

**Figure 4:**
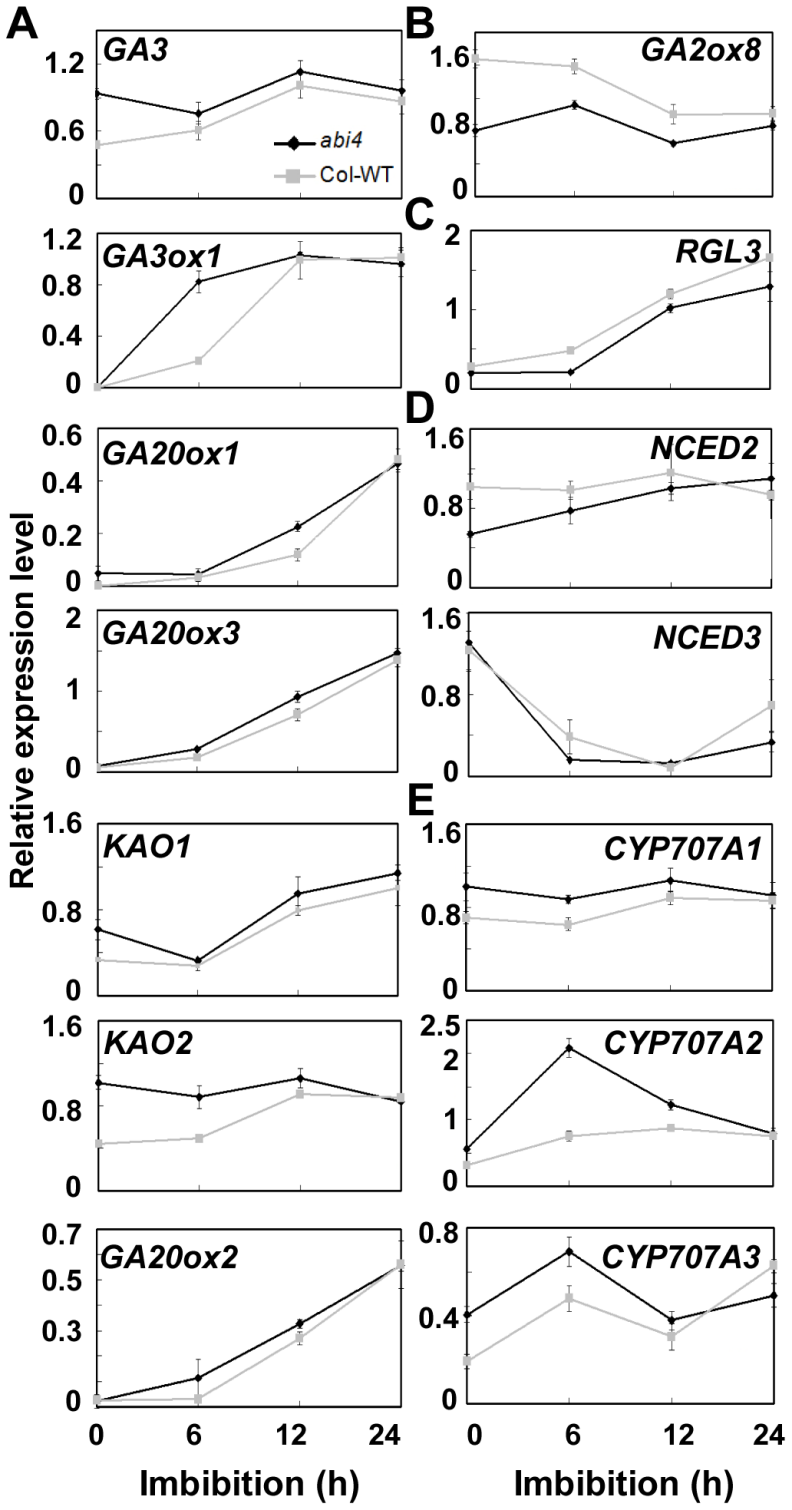
Gene expression analysis in dry and imbibed seeds. Gene expression was investigated by qRT-PCR during the course of the imbibition process. Two-week stored seeds were used for mRNA extraction, and three replications were performed. Primers used in the qRT-PCR assay are listed in Table S1. (A) GA biosynthesis genes. (B) GA catabolism genes. (C) *RGL3*, a negative regulator of GA signaling. (D) ABA biosynthesis genes. (E) ABA catabolism genes.

To further confirm that ABI4 functions as an attenuator of GA biogenesis during seed germination, we analyzed the effect of the *ABI4* mutation on the expression of GA biosynthesis genes and GA inactivation genes in dry and imbibed seeds. The results of qRT-PCR analysis showed that the transcript levels of GA biosynthesis genes, including *GA3*, *GA3ox1*, *GA20ox1*, *GA20ox3*, *KAO1*, *KAO2* and *GA20ox2*, were upregulated to varying degrees in *abi4* seeds after imbibition (Figure 4A). The expression levels of *GA3* and *KAO2* in dry *abi4* seeds were 2-fold higher than that in WT, and this trend was maintained throughout the imbibition treatment process (Figure 4A). Higher levels of *GA3ox1* mRNA were detected in the *abi4* mutant after 6 hours of imbibition (Figure 4A). The transcripts of *KAO1*, *GA20ox1*, *GA20ox2* and *GA20ox3* were higher in *abi4* than in WT during the entire imbibition process, although the differences were not significant (Figure 4A). By contrast, the transcript level of *GA2ox8*, a key GA inactivation gene, was lower in the *abi4* mutant than in the WT (Figure 4B). The increased expression of GA biosynthesis genes and the decreased expression of GA inactivation genes in imbibed seeds are accordance with the GA measurements in *abi4* mutant seeds which contains higher levels of active GA than the WT (Figure 3G). Consistent with this, the *RGL3* gene, which encodes a DELLA transcription regulator that represses testa rupture during seed germination [52], was downregulated in both dry and imbibed *abi4* seeds (Figure 4C).

Since ABA and GA regulate seed germination antagonistically [2], the expression levels of ABA biosynthesis and inactivation genes in dry and imbibed seeds were also analyzed. Analysis by qRT-PCR showed that the mRNA levels of ABA biosynthesis genes, including *NCED2* and *NCED3*, were downregulated in *abi4* (Figure 4D), while the ABA inactivation genes such as *CYP707A1*, *CYP707A2* and *CYP707A3* were upregulated (Figure 4E). The higher transcription levels of these three inactivation genes in *abi4* were maintained throughout the entire imbibition process. Notably, the expression level of *CYP707A2* in *abi4* was almost 4-fold higher than that in WT at 6 hours after imbibition (Figure 4E). The high transcription levels of ABA inactivation genes, and the low level of ABA synthesis in *abi4*, explain the results of ABA measurement (Figure 2). Together, the transcript levels of GA biosynthesis and ABA inactivation genes were upregulated in germinating *abi4* seeds, while GA inactivation and ABA biosynthesis genes were downregulated. These results are consistent with the notion that ABI4 negatively regulates GA biosynthesis while positively regulating ABA biogenesis (Figures 2, 3).

### ABI4 Binding to the Promoters of *CYP707A1* and *CYP707A2 In Vivo*

Previous studies have demonstrated that ABI4 is a versatile transcription factor that binds to the CACCG motif to promote the expression of some genes; this factor also binds to the CCAC element to directly inhibit the transcription of some genes [38], [50].

**Figure 5:**
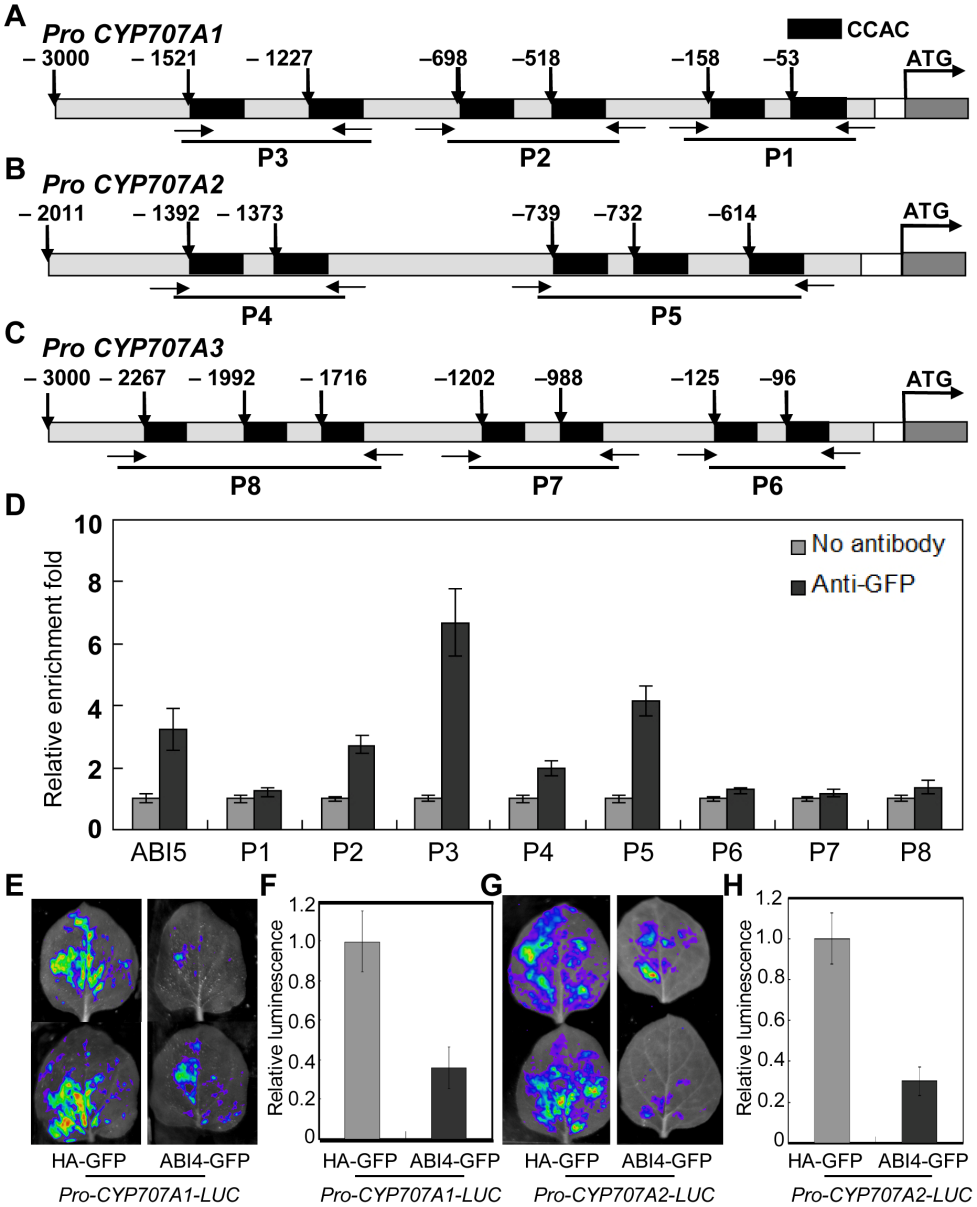
*ABI4* represses *CYP707A1* and *CYP707A2* expression by directly binding to these promoters. (A)–(C) Promoters of *CYP707A1* (A), *CYP707A2* (B) and *CYP707A3* (C) were analyzed. Fragments of 2,000–3,000 bp upstream of ATG were chosen as the promoter regions. (D) ChIP-qPCR was performed using specific primers corresponding to different promoter regions. *TUB4* was employed as an internal control, and the promoter of *ABI5* was used as a positive control. Primers used in the ChIP-qPCR assay are indicated by arrows and listed in Table S1. (E)–(H) Transient expression assay showed that ABI4 inhibits *CYP707A1* and *CYP707A2* transcription. Representative images of *N. benthamiana* leaves are shown in (E) and (G). Quantitative analyses of luminescence intensity are shown in (F) and (H). The experiments were performed three biological repeats and obtained the similar trend.

To investigate whether ABI4 directly regulates the expression some GA and ABA metabolism genes, we first examined the promoters of the genes described in Figure 4 because the expression levels of these genes were altered in *abi4* during germination. *CYP707A1*, *CYP707A2* and *CYP707A3* were most interesting because 6, 5 and 7 CCAC motifs were detected in these three promoters, respectively (Figure 5A, 5B and 5C). This inspired us to examine whether ABI4 directly binds to these promoters *in vivo*. We then conducted a ChIP (chromatin immunoprecipitation)-qPCR assay with the *ABI4* transgenic lines to examine whether ABI4 binds to these promoters directly. Because ABI4 binds directly to the promoter of *ABI5* [50], a DNA element of the *ABI5* promoter was used as positive control. Two independent *OE-ABI4* transgenic lines (OE1 and OE2) were subjected to ChIP-qPCR analysis, which produced similar results. We determined that the promoters of *CYP707A1* and *CYP707A2* were enriched in the chromatin immunoprecipitated DNA using the anti-GFP antibody (Figure 5D), especially the P2 and P3 regions in *CYP707A1* and the P5 region in *CYP707A2*. This result indicates that ABI4 directly binds to the promoters of *CYP707A1* and *CYP707A2 in vivo*. However, we did not detected significant enrichment of all of the elements tested from promoter of *CYP707A3* (Figure 5D), although this promoter possesses 7 CCAC motifs (Figure 5C). These results indicate that ABI4 may repress *CYP707A1* and *CYP707A2* expression by directly binding to the promoters of these genes.

#### ABI4 Inhibits *CYP707A1* and *CYP707A2* Transcription *In Vivo* and this Repression Effect is Dependent on the CCAC *cis*-element

The evidences from qPCR analysis in *abi4* and WT seeds (Figure 4E) and ChIP-qPCR (Figure 5D) indicated that ABI4 may possess the repression effect on the transcription of both *CYP707A1* and *CYP707A2* by directly binding to those promoters. Thus the transient expression system was employed to investigate whether ABI4 inhibits the expression of *CYP707A1* and *CYP707A2 in vivo*. By analyzing both of the promoters sequences (Figure 5A, 5B), the reporter plasmids Pro-*CYP707A1-LUC*, Pro-*CYP707A2-LUC* and the effector plasmid *pCanG-ABI4-GFP* were constructed separately. When the Pro-*CYP707A1-LUC* construct combined with *pCanG-HA-GFP* were performed, strongly LUC activity was detected (Figure 5E, 5F). However, when the *pCanG-HA-GFP* control vector was substituted by the equal amount of effector *pCanG-ABI4-GFP*, the LUC activity was decreased obviously (Figure 5E, 5F). Regarding the *CYP707A2* promoter, the similar trend also was detected (Figure 5G, 5H). All types of control were shown in Figure S5. These results revealed that ABI4 indeed inhibits both of the genes transcription activity *in vivo*.

Further, to check whether this repression effect of ABI4 depended on the CCAC motifs in the promoters, we mutated the CCAC motifs (changed to CCAA) in *CYP707A1* promoter. There are several CCAC motifs within the *CYP707A1* promoter, we chose the P3 region in which the binding capacity of ABI4 is the highest one among all elements analyzed (Figure 5D), and named the mutated CCAC motif as *m1* and *m2* in this P3 region, separately (Figure S6A). Using the transient expression system, our results showed that ABI4 could not inhibit *CYP707A1* transcription activity in the presence of any mutated promoter forms, *m1* (Figure S6B to S6D) or *m2* (Figure S6E to S6G), respectively, in contrast to the inhibition result of native promoter of *CYP707A1* (Figure 5E, 5F). Further, the similar trend was detected when we mutated the both of CCAC motifs (Figure S6H to S6J). Controls were provided in Figure S6K to S6M. These results revealed that the CCAC motifs in the *CYP707A1* promoter P3 region are important for the inhibition effect of ABI4.

### The *abi4* Rescues the Phenotypes of *ga1-t*, *cyp707a1* and *cyp707a2*

Physiological and molecular evidence support the notion that the biogenesis of ABA and GA during seed germination is affected by *ABI4*, and ABI4 positively regulates primary seed dormancy. To further confirm this conclusion, we subsequently dissected the genetic relationship between *ABI4* and hormone metabolism genes.

**Figure 6:**
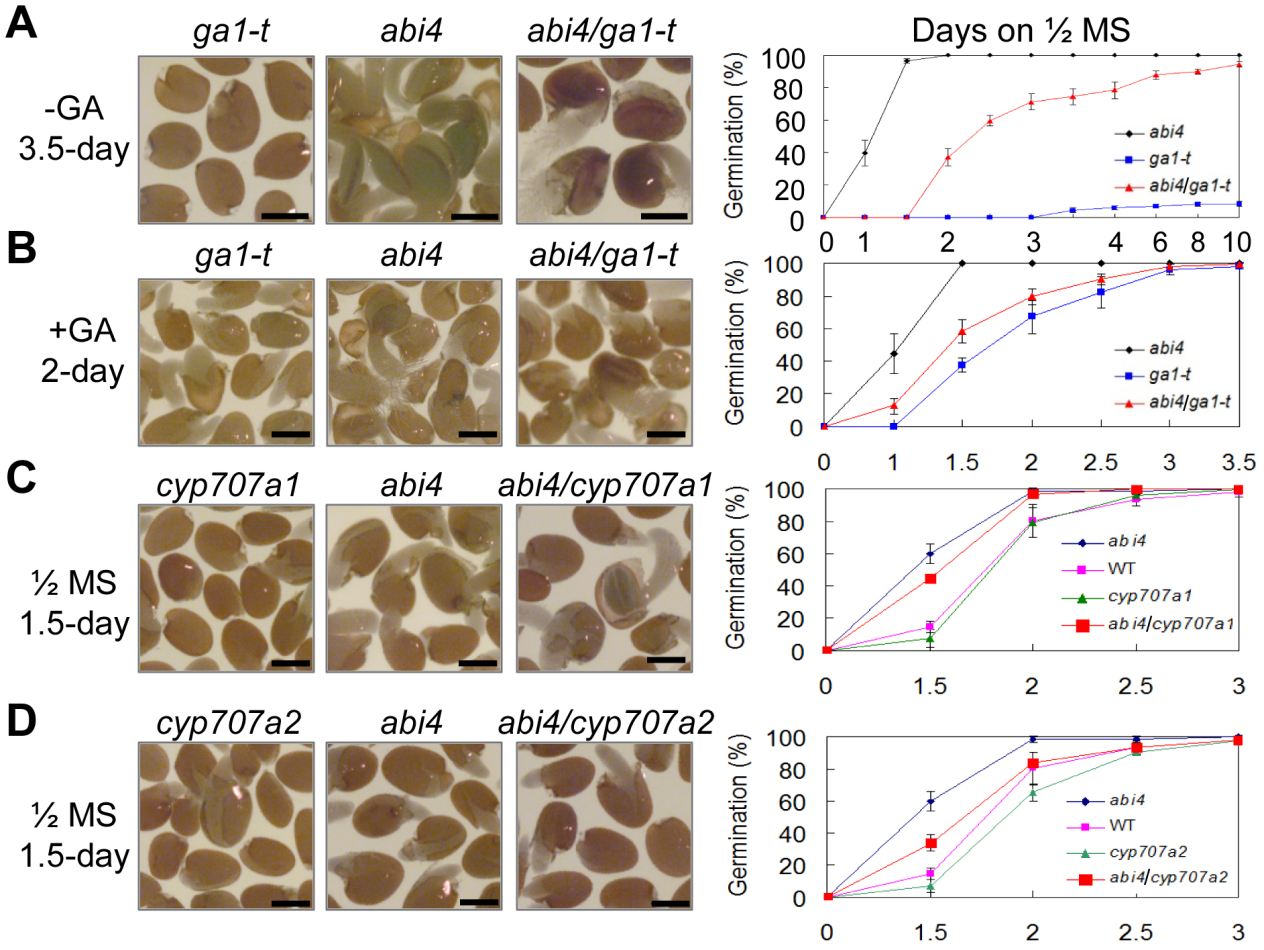
The *abi4* rescues the non-germination phenotype of *ga1-t* and restores the delayed germination of *cyp707a1* and *cyp707a2*. Quantitative analysis of germination rates are shown in the right panels (n≥45). One representative image (time points indicated in figures) per genotype is shown (left panels). Two-week stored seeds were used for analysis. Bar = 0.25 mm. Percentages are the average of three repeats ± standard error. (A) Seed germination of *abi4*, *ga1-t* and *abi4/ga1-t* in the absence of exogenous GA. (B) Seed germination of *abi4*, *ga1-t* and *abi4/ga1-t* in the presence of exogenous GA. (C) Seed germination of *abi4*, *cyp707a1* and *abi4/cyp707a1* mutants on 1/2 MS medium. (D) Seed germination of *abi4*, *cyp707a2* and *abi4/cyp707a2* mutants on 1/2 MS medium.

*GA1* encodes *ent*- ent-copalyl diphosphate synthase synthase, a key enzyme that catalyzes a relatively early biochemical reaction in the biosynthesis of GA [53], [54]. The *ga1* loss-of-function alleles cause GA deficiency and abolish seed germination in the absence of exogenous GA [26], [54]. *OE-ABI4* seeds were sensitive to PAC during germination, while *abi4* seeds were resistant (Figure 3E), and further, the GA biogenesis was attenuated in *abi4* seeds compared to WT (Figure 3G), indicating that GA biosynthesis is indeed negatively regulated by ABI4. Therefore, we examined whether *abi4* could rescue the non-germination phenotype conferred by *ga1-t*. First, we created a double mutant between the *abi4* and *ga1-t* homozygous mutants through genetic crossing. Subsequently, seed germination was analyzed in the *abi4*, *ga1*-*t* and *abi4*/*ga1-t* double mutants using seeds subjected to two weeks of dry storage. The results showed that the *abi4*/*ga1-t* double mutants germinated normally, and the cotyledons greened normally, even in the absence of exogenous GA, while *ga1*-*t* did not germinate under these condition (Figures 6A, S7A). As expected, the exogenous application of GA restored the germination of *ga1*-*t*. Furthermore, the *abi4*/*ga1-t* double mutant also germinated, and the cotyledons greened slightly more quickly than those of *ga1*-*t* in the presence of exogenous GA (Figures 6B, S7B). These results demonstrate that ABI4 indeed negatively regulates GA biogenesis from the view of genetics.

On the other hand, the *cyp707a1* and *cyp707a2* mutants accumulate higher levels of ABA than the WT and thus exhibit the delayed germination phenotype [15]. Since the ABA level was downregulated in the *abi4* mutant (Figure 2) and ABI4 directly inhibits *CYP707A1* and *CYP707A2* expression by binding to those promoters (Figure 5D to 5H), we tested whether *abi4* could rescue the germination defect phenotype of *cyp707a1* and *cyp707a2*. Therefore, *abi4/cyp707a1* and *abi4/cyp707a2* double mutants were created between *abi4* and the homozygous mutant *cyp707a1* (SALK_069127) and *cyp707a2* (SALK_083966C), respectively. Our results showed that the seeds of these double mutants showed higher germination frequencies than the corresponding *cyp* single mutants, *cyp707a1* and *cyp707a2*, but lower than *abi4* when the seeds were subjected to two weeks of dry storage (Figure 6C, 6D, right panel). Given that ABI4 positively regulates ABA biogenesis, we speculated that the reason responsible for the recovery of *abi4/cyp707a1* and *abi4/cyp707a2* regarding the delayed germination phenotype of *cyp707a2* and *cyp707a2* is that the ABA biogenesis is impaired in these double mutants. For this end, we further detected the ABA content in the *cyp707a2* single mutant and *abi4/cyp707a2* double mutant respectively. Indeed, our results revealed that the ABA level in *cyp707a2* is significantly increased compared to WT (Figure S8), which is consistent with the previous study [15]. Importantly, we detected that the ABA content in *abi4/cyp707a2* is decreased compared to *cyp707a2* single mutant (Figure S8). These results indicate that a mutation in the *ABI4* locus recovers the reduced germination potential of *cyp707a1* and *cyp707a2* through attenuating the ABA biogenesis. Together, these genetic analyses between *CYP707A1*, *CYP707A2*, *GA1* and *ABI4* further confirmed the notion that ABI4 indeed positively regulates ABA biosynthesis and negatively regulates GA biogenesis.

## Discussion

Physiological analysis of germination, hormone measurements, gene expression analysis and biochemical and genetic analysis have demonstrated that a mutation in the *ABI4* locus indeed reduces primary seed dormancy, and the molecular mechanism responsible for this phenotype is as follows: ABA biogenesis is downregulated, and GA biosynthesis is upregulated in the *abi4* mutants. The present study clarifies that like ABI3, ABI4 also positively regulates primary seed dormancy. Further, this study also strongly suggests and opens up the possibility that ABI4 plays pivotal and complex roles in the crosstalk between ABA and GA in the regulation of primary seed dormancy and early plant development.

### ABI4 Positively Regulates Primary Seed Dormancy

Pre-harvest sprouting of diverse cereal seeds usually occurs under humid conditions during harvest time and results in the germination of grains that are still on the mother plant. Sprouting, which results from the reduced dormancy level of crop seeds, lowers the value of crop seeds in terms of both quantity and quality [2], [6]. Therefore, pre-harvest sprouting has attracted increasing amounts of attention from researchers, especially in agronomic regions; the precise molecular mechanism underlying seed dormancy and pre-harvest sprouting is worth exploring.

In the present study, the *abi4* seeds obviously germinated significantly more quickly than WT when the seeds were subjected to short-term storage; this mutant even exhibited the vivipary phenotype (Figure 1). On the other hand, it is noteworthy that the percentages of germination of *abi4* and WT seeds were comparable at 4.5 days after sowing (all reached nearly 100%; Figure 1A to 1D), which is in accordance with previous result [23]. In a previous study, the germination rate was scored at 5 days after sowing, and the *abi4* mutant showed the same degree of dormancy as WT seeds (both genotypes reached 100% germination) [23]. Therefore, ABI4 was thought to have no effect on seed dormancy. Subsequent studies and reviews cited this conclusion [3], [31], [43]. Recently, two studies showed that CHOTTO1 regulates primary seed dormancy positively, and, more interestingly, *ABI4* likely acts in the same genetic pathway as *CHOTTO1* [10], [44]. Both studies, along with our own occasionally observation that about *abi4* germinated more quickly than WT when the siliques fell onto the soil, inspired us to reconfirm the effect of ABI4 on primary seed dormancy. We speculate that the reason for the previous conclusion (that ABI4 has no effect on seed dormancy) is that the germination rates were not scored using detailed time-course analysis [23].

Seed dormancy can be classified as primary or secondary seed dormancy [55]. Freshly harvested seeds, or dormant seeds subjected to short-term storage, are deemed to have primary dormancy, which is induced by ABA during seed maturation on the mother plant and is abolished by longer period of dry-storage treatment (after-ripening) [10], [56], [57]. By contrast, secondary dormancy can be induced in seeds with non-deep physiological dormancy after seed dispersal, and it is often associated with annual dormancy cycles in seed banks [56]. In the present study, *abi4* seeds subjected to shorter period of dry-storage showed reduced seed dormancy levels and even the vivipary phenotype (Figure 1A to 1D, 1G). By contrast, the germination frequencies of *abi4* and WT were comparable when the seeds subjected to longer period of storage (Figure 1E, 1F). On the other hand, further investigation revealed that *OE-ABI4* seeds subjected to two weeks of storage germinated more slowly than WT seeds (Figure 3D), and the cotyledon greening rates of different genotypes were consistent with the dormancy levels (Figure S4A). Taken together, we conclude that ABI4 indeed positively regulates primary seed dormancy.

### ABI4 Positively Regulates ABA Biogenesis and Negatively Regulates GA Biosynthesis during Seed Germination

After confirming the effect of ABI4 on primary seed dormancy, we dissected the molecular mechanism underlying this phenotype. The reduced primary seed dormancy of *abi4* was moderately decreased by stratification and was even abolished by longer period of after-ripening treatment (Figure 1B, 1D, 1E, 1F). Furthermore, stratification and after-ripening treatments reduce ABA content [10], [49]. Therefore, we tested the ABA levels in dry and imbibed seeds. As expected, the ABA content in dry *abi4* seeds was lower than that in WT and became comparable after stratification (Figure 2). This result is similar to previously reported results about CHOTTO1, which also positively regulates primary seed dormancy [10], [44]. In these studies, the ABA level was downregulated in the *cho1* mutant, which was responsible for the reduced primary seed dormancy phenotype of *cho1* [10]. Therefore, we conclude that the decreased ABA level in the *abi4* mutant is at least partially responsible for the reduced primary seed dormancy phenotype, and further, ABI4 positively regulates ABA biogenesis. On the other hand, GA biosynthesis is enhanced in the ABA deficient *aba2* mutant, indicating that ABA is involved in the suppression of GA biosynthesis in both developing and imbibed seeds [9]. Because the ABA content in *abi4* seeds was markedly downregulated (Figure 2), we tested the responses of *abi4* and *OE-ABI4* to PAC and GA during seed germination. A previous report showed that the increased resistance to PAC suggests that the mutant contains higher levels of active GA or stronger GA signaling than the WT [51]. We found that *OE-ABI4* was sensitive to PAC and *abi4* was resistant, while exogenous GA rescued the delayed germination phenotype of *OE-ABI4* (Figures 3D to 3F, S4), and further, the GA measurements result showed that *abi4* seeds indeed contain higher levels of active GA_4_ than the WT (Figure 3G). These results are consistent with the ABA measurements (Figure 2). Therefore, we propose that ABI4 attenuates GA biosynthesis and promotes ABA biosynthesis to precisely regulate seed germination.

To further confirm the changes in ABA and GA content during seed germination, we also investigated the expression levels of ABA and GA biosynthetic and inactivation genes in dry and imbibed seeds. The results showed that the expression of most genes involved in ABA and GA metabolism was altered in dry and imbibed *abi4* seeds (Figure 4), which is consistent with the results of ABA and GA quantification, and the analysis of the responsiveness of *OE-ABI4* and *abi4* to GA and PAC treatments (Figures 2, 3, S4). These results were similar to results obtained from the analysis of sorghum grains, i.e., changes in the expression level of GA metabolism genes affects the seed dormancy and germination potential of sorghum grains [58]. In particular, the expression levels of *CYP707A1* and *CYP707A2* were remarkably decreased in the *abi4* seeds (Figure 4E). Furthermore, ChIP-qPCR analysis and the tobacco transient expression assays revealed that ABI4 inhibits both of the two ABA inactivation genes (*CYP707A1* and *CYP707A2*) expression by directly binds to the promoters (Figure 5D). In addition, the CCAC motifs in these promoters are important and the inhibition effect of ABI4 on its transcription was depended on the CCAC *cis*-element (Figure S6).

Further evidence confirming the regulation of ABA biogenesis by ABI4 was obtained by genetic analysis; the *abi4* mutant rescued the delayed germination phenotype of *cyp707a1* and *cyp707a2* (Figure 6C, 6D). Accordingly, our further experimental evidences demonstrated that ABI4 directly repress *CYP707A1* and *CYP707A2* expression to promote ABA biosynthesis (Figure 5E to 5H), and the higher expression level of *CYP707A1* and *CYP707A2* in the absence of *ABI4* result in reduced ABA content and, subsequently, the decreased seed dormancy level (Figures 2, 5). Notably, except for *cyp707a1* and *cyp707a2*, *abi4* also rescued the non-germination phenotype of *ga1-t* without exogenous GA treatment (Figure 6A), suggesting that ABI4 is indeed involved in regulation of GA biogenesis. Mutation at early stage of GA synthesis gene does not totally abolish GA in plant, and the *ga1-3*, an allele mutant of *ga1-t*, contains very low level of GA [59]. In *abi4* and *abi4*/*ga1-t* double mutants, reduced ABA contents and activated downstream GA synthesis and down regulated GA metabolic gene transcription might increase GA/ABA ratio in seeds, thus promotes the germination of *abi4*/*ga1-t* double mutant (Figures 4, 6A). *abi4* has the similar effects of *spy*, *rgl2* and *abi3* on the *ga1* mutant [22], [28], [29]; these genes also are negative regulators of the GA biogenesis or signaling pathway. Taken together, we conclude that ABI4 regulates ABA biogenesis positively, and GA biosynthesis negatively, during seed germination.

Previous elegant studies demonstrated that ABI4 is a key ABA signaling component per se [31], and in this study, we further showed that ABI4 is also involved in ABA and GA biogenesis (Figures 2, 3). High GA level could induce the transcription of α-amylase gene, whose product in turn hydrolyzes the seed coat which is essential for normal germination process. In opposite, ABA inhibits seed germination through suppressing the α-amylase gene expression [3]. Furthermore, previous study revealed that ABA is involved in the suppression of GA biogenesis [9]. Therefore, the decreased ABA level in *abi4* seeds could further activates the GA biogenesis, and subsequently, the increased GA content further promotes the α-amylase gene transcription. Accordingly, the seed dormancy level of *abi4* is decreased.

### ABI4 Positively Regulates ABA Signaling during Seed Germination

Although the decreased ABA level and increased GA content in *abi4* seeds are responsible for the reduced primary seed dormancy in this mutant (Figures 2, 3, S4), it is noteworthy that reduced seed dormancy was also detected when the short-term stored *abi4* seeds were stratified (Figure 1B, 1D), even the corresponding ABA levels were comparable between *abi4* and WT after stratification treatment (Figure 2). These results suggest that ABA signaling plays an important role in the control of primary seed dormancy. Indeed, previous studies have demonstrated that ABI4 positively regulates ABA signaling during seed germination [31], [60], and our results are consistent with this conclusion (Figure 1D). The other evidence about the key regulators in ABA signaling involved in seed dormancy control was from the analysis of the mutation in *ABI3* locus. Similar to *abi4*, *abi3* also was found to show the decreased seed dormancy [18]. ABI3, ABI4 and ABI5 were demonstrated to work in the same pathway in ABA signaling. Whether ABI5 is also involved in seed dormancy still need to be addressed in the future. Therefore, ABA signaling might also play a positive role during the control of seed dormancy.

Taken together, the present study demonstrates that ABI4 positively regulates primary seed dormancy by mediating the biogenesis of ABA and GA. Further, this study also strongly suggests that ABI4 plays a pivotal role in these two signaling pathways. Further functional dissection of ABI4 during the biosynthesis and signaling of ABA and GA is necessary to obtain a deeper understanding of the crosstalk between these two hormones.

## Methods

### Plant Materials and Growth Conditions

Arabidopsis ecotype Columbia-0 was used as the wild type in this study. The point mutant *abi4-1* (CS8104) and the T-DNA insertion mutants *abi4-t* (SALK_080095), *cyp707a1* (SALK_069127) and *cyp707a2* (SALK_083966C) were obtained from the ABRC (The Ohio State University, Columbus, OH, USA). It is noted that the T-DNA insertion mutant SALK_080095 was named as *abi4-2* [47]. But the name of *abi4-2* has been given much earlier to the other mutant harboring a point mutant in *ABI4* gene [35].Thus the T-DNA insertion line SALK_080095 was named as *abi4-t* in this work. The *ga1-t* mutant (SALK_023192) in the Columbia-0 background was a gift from Dr. Xiangdong Fu (Institute of Genetics and Developmental Biology, Chinese Academy of Sciences, Beijing). The *abi1-1*, *abi2-1*, *snrk2.2/snrk2.3* mutants seeds were supplied by Dr. Zhizhong Gong (College of Biological Sciences, China Agricultural University, Beijing). Arabidopsis seeds were surface-sterilized with 10% bleach and washed at least four times with sterile water. Sterile seeds were suspended in 0.2% agarose and sown on 1/2 MS medium plus 1% sucrose. The seeds were stratified on plates in the dark at 4°C for 0 or 3 days, depending on the experiment, and then transferred to a tissue culture room at 22°C under a 16-h-light/8-h-dark photoperiod. For *ga1-t*, the seeds were soaked in 100 µM GA solution for 3 days at 4°C, as the *ga1-t* mutant cannot germinate in the absence of exogenous GA. Normal 1/2 MS medium was supplemented with 1% sucrose and, unless otherwise noted, GA (product number G7645, Sigma-Aldrich Company ltd, USA) or PAC (product number 46046, Sigma-Aldrich Company ltd, USA) was added as needed.

### Generation of Transgenic Plants

Transgenic plants carrying constitutively expressing *ABI4* were generated. To produce *35S-ABI4* plants, the 987-bp CDS (coding sequence) fragment was amplified by PCR and then cloned into the vector *pCanG-HA-GFP*, in which *ABI4* was expressed under the control of the CaMV 35S promoter. Transformation of Arabidopsis was performed by the vacuum infiltration method using the *Agrobacterium tumefaciens* strain EHA105 [61]. T_2_ seeds were germinated on MS plates containing 50 mg/mL kanamycin for vector *pCanG-HA-GFP*, and the resistant seedlings were transferred to soil to obtain homozygous T_3_ seeds. For more detailed phenotypic analysis, two independent T_3_ homozygous lines containing a single insertion were employed.

### Seed Germination, Photography and Vivipary Testing

To test germination rates, seeds were collected at the same time. Seeds subjected to various periods of dry storage were sown on normal 1/2 MS medium or 1/2 MS medium supplemented with various concentrations of GA or PAC. Radicle emergence was scored at the indicated time points, and at the same time, the percentages of cotyledon greening were also scored. For each germination test, approximately ≥45 seeds per genotype were used, and three experimental replications were performed. The average germination percentage ± SE (standard error) of triplicate experiments was calculated. For photography, a Leica MZ16 FA stereomicroscope was employed (Leica Company, Germany). Photographs were taken using the same settings at the indicated time points. The vivipary assay was performed according to a previously described protocol [51]. Developing siliques at the long-green stage were collected from the same sites of plants with various genotypes, sterilized with 70% ethanol for 1 minute and 25% bleach for 10 minutes and plated on 1/2 MS medium or damp soil.

### Gene Expression Analysis

Total RNA preparation (from dry or imbibed seeds at various times), first-strand cDNA synthesis and qRT-PCR were performed as previously described [62]. DNase I-treated total RNA (2 µg) was denatured and subjected to reverse transcription using Moloneymurine leukemia virus reverse transcriptase (200 units per reaction; Promega Corporation). Quantitative PCR was performed using the SsoFast EvaGreen Supermix (Bio-Rad) and a CFX96 Touch Real-Time PCR Detection System (Bio-Rad). Gene expression was quantified at the logarithmic phase using the expression of the housekeeping *18S* RNA as an internal control. Three biological replicates were performed for each experiment. Primer sequences for qRT-PCR are shown in Table S1.

### Protein Extraction and Protein Gel Blot Analysis

To test the ABI4 protein levels in transgenic plants (*35S-ABI4-GFP*), western blotting was performed according to previously described protocols [62], [63]. Approximately two-week-old seedlings grown on 1/2 MS medium were ground in liquid nitrogen and extracted with 4 M urea buffer. Crude extracts were separated by SDS-PAGE and transferred onto nitrocellulose membranes. The membranes were stained with 0.2% Ponceau S, with Rubisco serving as an internal control. The anti-GFP antibody was purchased from Santa Cruz Biotechnology, Inc.

### Chromatin Immunoprecipitation (ChIP)-qPCR Assay

ChIP was performed as previously described [64], with minor modifications. Transgenic seeds containing *35S-ABI4-GFP* were grown on 1/2 MS medium for approximately 2 weeks. The seedlings were then harvested (1.5 g) and crosslinked with 1% formaldehyde for 30 minutes under a vacuum; the crosslinking was stopped with 0.125 M glycine. The seedlings were ground in liquid nitrogen, and the nuclei were isolated. Immunoprecipitations were performed with the anti-GFP antibody and protein G beads. Immunoprecipitation in the absence of anti-GFP served as the control (CK). DNA was precipitated by isopropanol, washed with 70% ethanol and dissolved in 10 µl water containing 20 µg/mL RNase. The qRT-PCR analysis was performed using specific primers corresponding to different promoter regions of *CYP707A1*, *CYP707A2* and *CYP707A3*. *TUB4* was used as an internal control. Since ABI4 directly binds to the promoter of *ABI5* [50], this promoter was employed as a positive control. Primers used in the ChIP-qPCR assay are shown in Table S1.

### Analysis of *CYP707A1* and *CYP707A2* Promoters Activity by *ABI4 In Vivo*

This transient expression assay was performed in *N. benthamiana* leaves as previously described [65]. The 2329 bp for native *CYP707A1* promoter (Pro-*CYP707A1*) and 2015 bp for native *CYP707A2* (Pro-*CYP707A2*) were amplified separately from genomic DNA. In addition, the several mutated *CYP707A1* promoter fragments (including Pro-*CYP707A1 (m1)*, Pro-*CYP707A1 (m2)*, Pro-*CYP707A1 (m1+m2)*) were generated by PCR amplification. All these five promoter fragments were cloned into *pENTR* using the pENTR Directional TOPO cloning kit (Invitrogen). Then, these promoter versions were fused with the luciferase reporter gene *LUC* through the Gateway reactions into the plant binary vector *pGWB35* [66]to generate the several reporters constructs. The effector construct was the *pCanG-ABI4-GFP*.

### Quantification of ABA

For analysis of ABA content in dry or imbibed seeds, the seeds were ground in liquid nitrogen, and 150 mg of seed powder was homogenized and extracted for 24 h in methanol containing D6-ABA (purchased from OIChemIm Co. Ltd.) as an internal standard. Purification was performed with an Oasis Max solid phase extract cartridge (150 mg/6 cc; Waters) and eluted with 5% formic acid in methanol. The elution was dried and reconstituted, and it was then injected into a liquid chromatography–tandem mass spectrometry system consisting of an Acquity ultra performance liquid chromatograph (Acquity UPLC; Waters) and a triple quadruple tandem mass spectrometer (Quattro Premier XE; Waters). Three biological replications were performed.

### Quantification of Endogenous Gibberellins

The endogenous gibberellins were determined by the method described [67]. Arabidopsis seeds (200 mg) were frozen in liquid nitrogen, ground to fine powder, and extracted with 80% (v/v) methanol. GA isotope standards were added to plant samples before grinding. The crude extracts were purified by reversed-phase solid-phase extraction, ethyl ether extraction and derivatization. The resulting mixture was injected into capillary electrophoresis-mass spectrometry (CE-MS) for quantitative analysis.

## Supporting Information

Figure S1 Quantitative analysis of cotyledon greening rates of *abi4*and WT. (A)–(F) Cotyledon greening rates of WT and *abi4* on 1/2 MS medium with or without stratification treatment are shown. Seeds were stored for 1 or 2 weeks or 6 months after harvest and used for analysis. Percentages are the average of three repeats ± standard error.
(TIF)

Figure S2 *abi4-t* confirms the reduced primary seed dormancy phenotype of *abi4*. Quantitative analysis of germination rates (A) and cotyledon greening rates (B) of *abi4-t* and WT on 1/2 MS medium are shown. Percentages are the average of three repeats ± standard error. One-week stored seeds were used.
(TIF)

Figure S3 Comparison of the decreased primary seed dormancy phenotype of *snrk2.2/snrk2.3*, *abi4* and *abi1-1*, *abi2-1*. (A) Quantitative analysis of germination of WT, *abi4* and *snrk2.2/snrk2.3* seeds on 1/2 MS medium without stratification treatment. Freshly harvested seeds were used for analysis. Percentages are the average of four repeats ± standard error. (B) Quantitative analysis of germination of Ler, *abi1-1* and *abi2-1* seeds under the same experimental conditions (without stratification). Seeds were stored for 6 months after harvest and subjected to analysis. Percentages are the average of four repeats ± standard error.
(TIF)

Figure S4 Responses of *abi4* and *OE-ABI4* to GA and PAC treatment at the postgerminative growth stage. Cotyledon greening rates of WT, *abi4*, OE1 and OE2 were scored on 1/2 MS medium (A), 1/2 MS medium supplemented with 15 µM PAC (B) and 1/2 MS medium supplemented with 0.5 µM GA (C). Quantitative analysis of germination rates are shown in the right panels (n≥45). One representative image (time points indicated in figures) per genotype is shown (left panels). Bar = 0.25 mm. Percentages are the average of three repeats ± standard error.
(TIF)

Figure S5 Internal controls for the tobacco transient expression assay. (A) and (B) qRT-PCR analysis of *GFP* expression in the infiltrated leaf areas. Total RNAs were extracted from leaves of *N. benthamiana* infiltrated with the *pCanG-HA-GFP* or *pCanG-ABI4-GFP* combined with *Pro-CYP707A1-LUC* (A) or *Pro-CYP707A2-LUC* (B) constructs. Tobacco *Actin* was employed as the internal control in the qRT-PCR analysis. The experiments performed three biological repeats and obtained the similar trend. (C) and (D) using the PCR-DNA amount of promoters to represent the equal plasmid DNA in agro-infiltration were applied between parallel experiments in Figure 5.
(TIF)

Figure S6 ABI4 could not inhibit *CYP707A1* transcription when the CCAC *cis*-elements were mutated. (A) Mutated scheme of promoter of *CYP707A1*. The two CCAC motifs in P3 region were changed to CCAA. (B)–(M) Different mutated forms of *CYP707A1* promoter were analyzed. Representative images of *N. benthamiana* leaves are shown in (B), (E) and (H). The corresponding quantitative analyses of luminescence intensity are shown in (C), (F) and (I). (D), (G) and (J) represented the *GFP* expression in the infiltrated tobacco leaves for the different combinations. Total RNAs were extracted from leaves of *N. benthamiana* leaves. The experiments performed three biological repeats and obtained the similar trend. Tobacco *Actin* was employed as the internal control in qRT-PCR analysis. (K) to (M) Using the PCR-DNA amount of promoters to represent the equal plasmid DNA in agro-infiltration were applied between parallel experiments. (B)–(D) For Pro-*CYP707A1 (m1)*-LUC. (E)–(G) For Pro-*CYP707A1 (m2)*-LUC. (H)–(J) For Pro-*CYP707A1 (m1+m2)*-LUC.
(TIF)

Figure S7 Cotyledon greening rates of *abi4*, *ga1-t* and *abi4/ga1-t* with or without exogenous GA treatment. Quantitative analysis results were shown in the right panels (n≥45). One representative image (time points indicated in figures) per genotype is shown (left panels). Bar = 0.25 mm. Percentages are the average of three repeats ± standard error. (A) Cotyledon greening of *abi4*, *ga1-t* and *abi4/ga1-t* mutants in the absence of exogenous GA treatment. (B) Cotyledon greening of *abi4*, *ga1-t* and *abi4/ga1-t* mutants in the presence of exogenous GA treatment.
(TIF)

Figure S8 Endogenous ABA measurements in different genotypes. Endogenous ABA levels in WT, *cyp707a1* and *abi4/cyp707a1* seeds were quantified. Two-week stored seeds were used for analysis.
(TIF)

Table S1 Primer sequences used in this study.
(DOC)
