# Supplementary figures and images for: ABI4 Regulates Primary Seed Dormancy by Regulating the Biogenesis of Abscisic Acid and Gibberellins in Arabidopsis
Source: PLoS Genet. 2013 Jun 20;9(6):e1003577. doi: 10.1371/journal.pgen.1003577 (PMC3688486; doi:10.1371/journal.pgen.1003577)

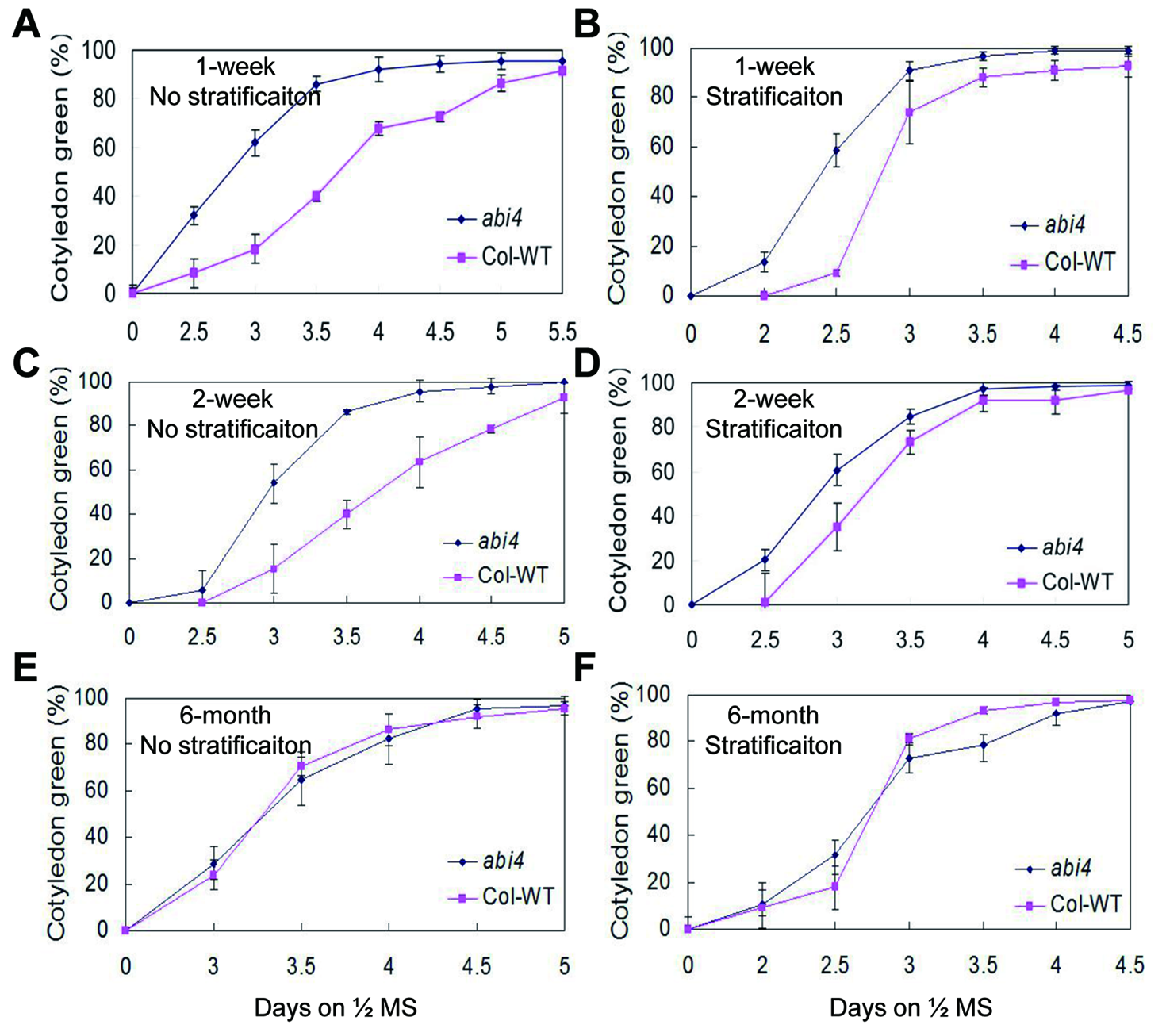

Supplement: Figure S1 — Quantitative analysis of cotyledon greening rates of abi4and WT. (A)–(F) Cotyledon greening rates of WT and abi4 on 1/2 MS medium with or without stratification treatment are shown. Seeds were stored for 1 or 2 weeks or 6 months after harvest and used for analysis. Percentages are the average of three repeats ± standard error. (TIF) [file pgen.1003577.s001.tif]

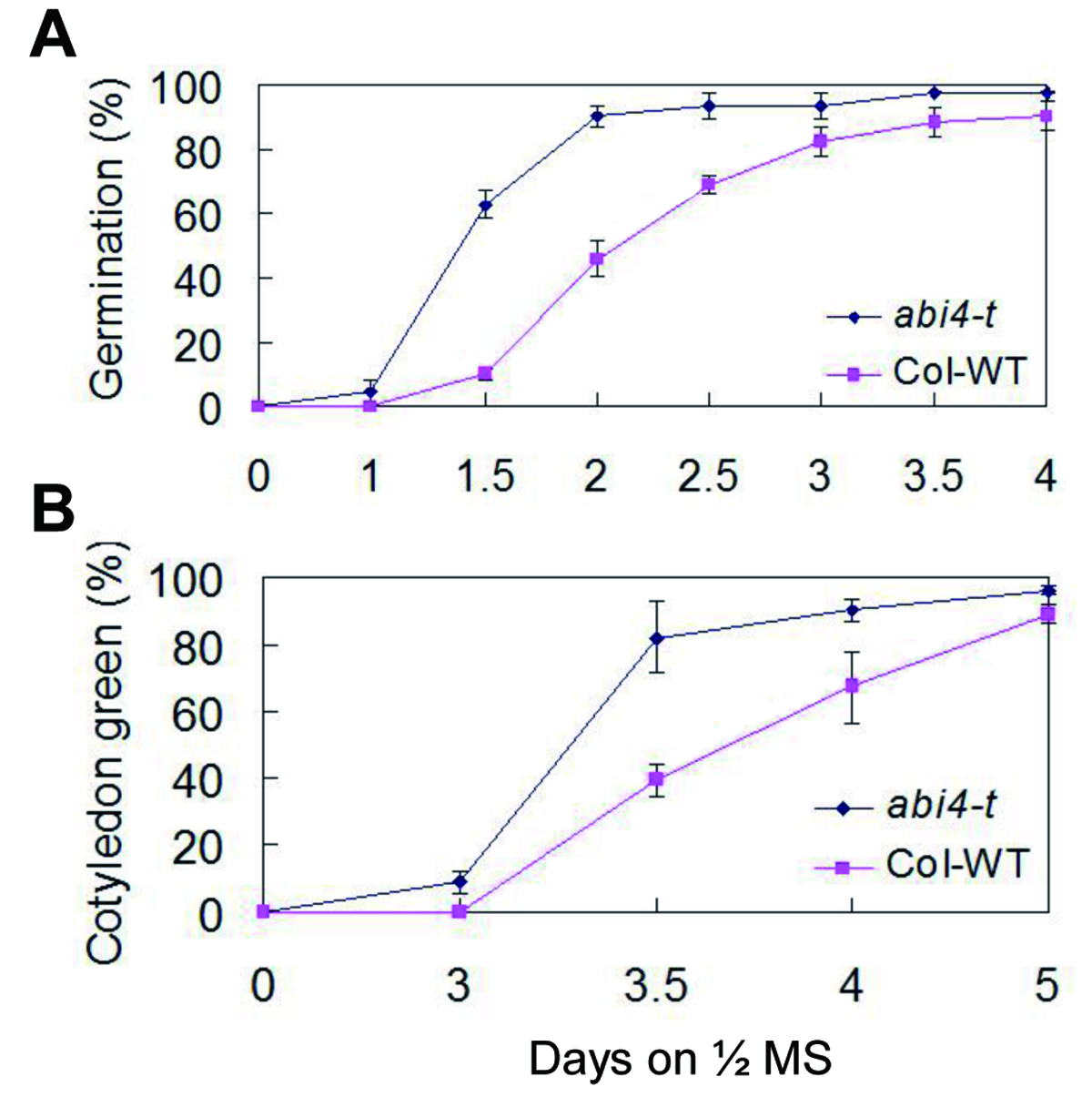

Supplement: Figure S2 — abi4-t confirms the reduced primary seed dormancy phenotype of abi4. Quantitative analysis of germination rates (A) and cotyledon greening rates (B) of abi4-t and WT on 1/2 MS medium are shown. Percentages are the average of three repeats ± standard error. One-week stored seeds were used. (TIF) [file pgen.1003577.s002.tif]

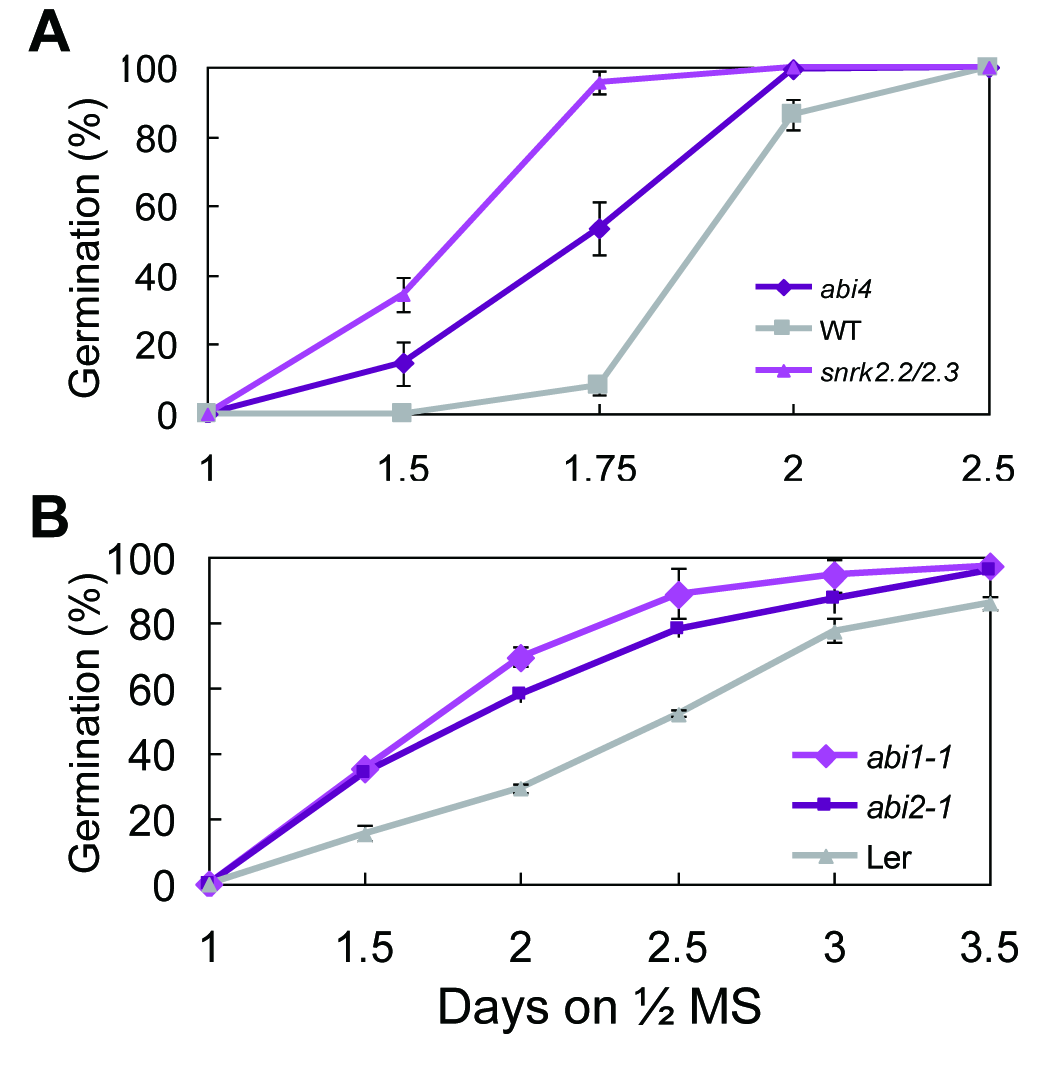

Supplement: Figure S3 — Comparison of the decreased primary seed dormancy phenotype of snrk2.2/snrk2.3, abi4 and abi1-1, abi2-1. (A) Quantitative analysis of germination of WT, abi4 and snrk2.2/snrk2.3 seeds on 1/2 MS medium without stratification treatment. Freshly harvested seeds were used for analysis. Percentages are the average of four repeats ± standard error. (B) Quantitative analysis of germination of Ler, abi1-1 and abi2-1 seeds under the same experimental conditions (without stratification). Seeds were stored for 6 months after harvest and subjected to analysis. Percentages are the average of four repeats ± standard error. (TIF) [file pgen.1003577.s003.tif]

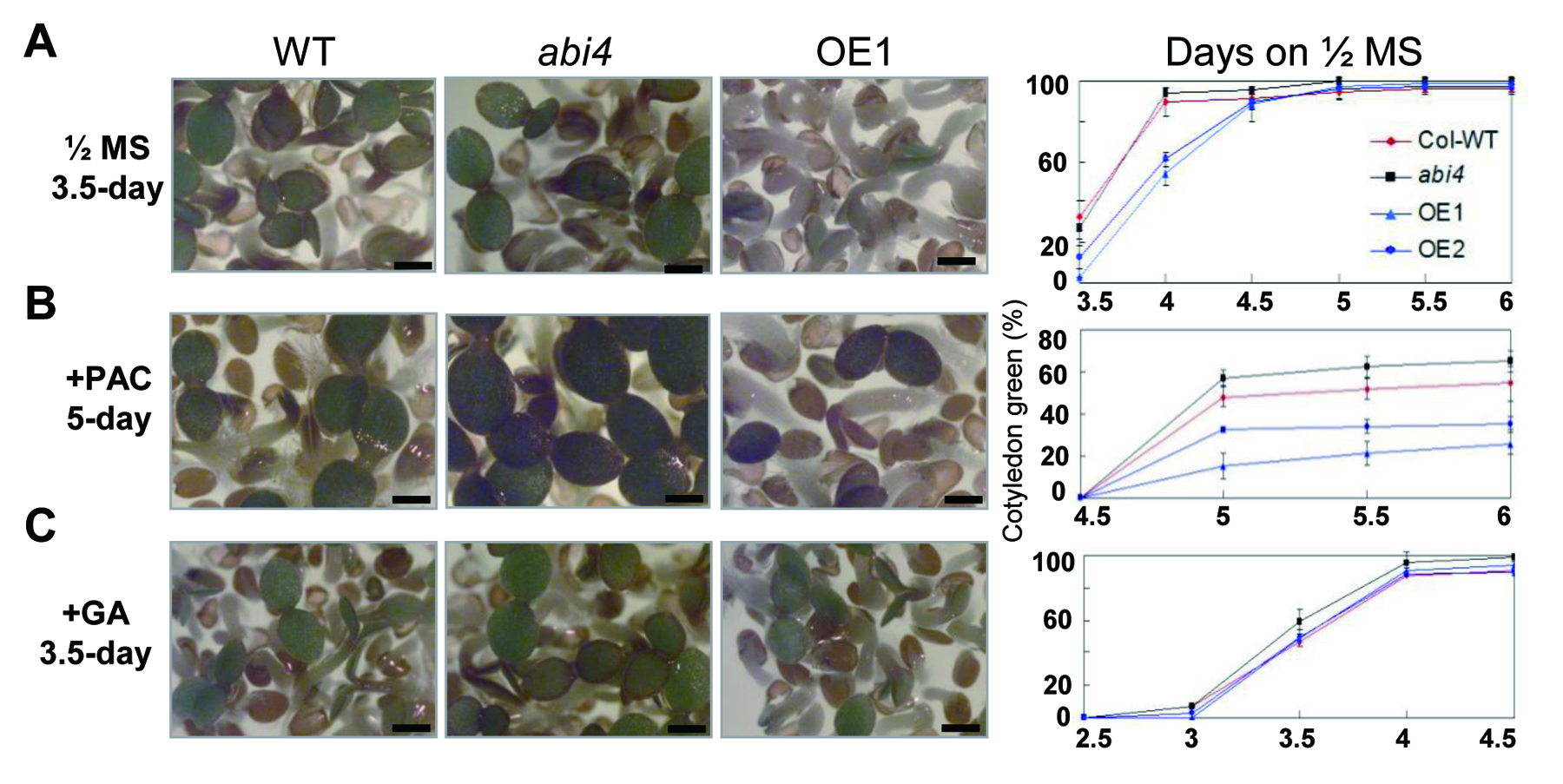

Supplement: Figure S4 — Responses of abi4 and OE-ABI4 to GA and PAC treatment at the postgerminative growth stage. Cotyledon greening rates of WT, abi4, OE1 and OE2 were scored on 1/2 MS medium (A), 1/2 MS medium supplemented with 15 µM PAC (B) and 1/2 MS medium supplemented with 0.5 µM GA (C). Quantitative analysis of germination rates are shown in the right panels (n≥45). One representative image (time points indicated in figures) per genotype is shown (left panels). Bar = 0.25 mm. Percentages are the average of three repeats ± standard error. (TIF) [file pgen.1003577.s004.tif]

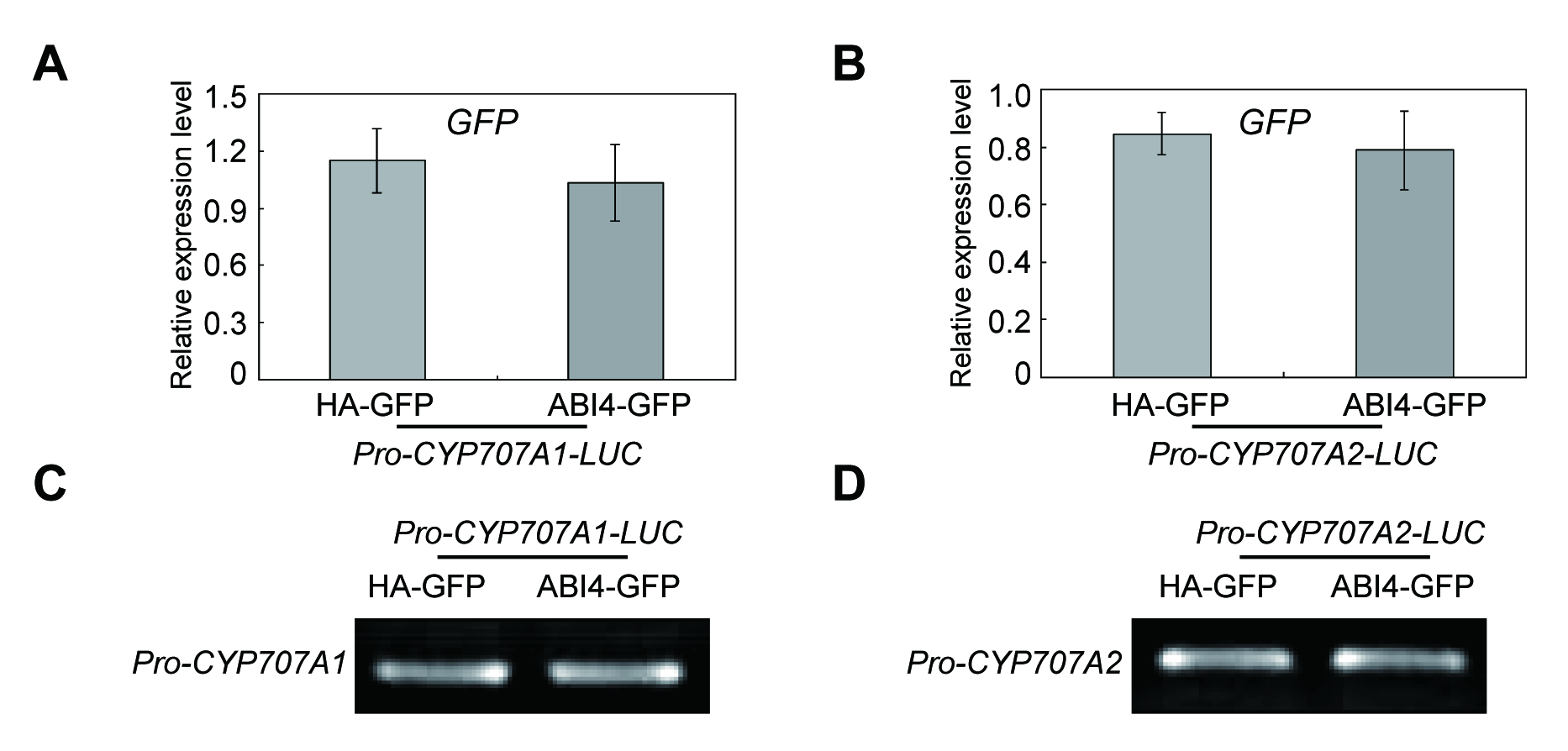

Supplement: Figure S5 — Internal controls for the tobacco transient expression assay. (A) and (B) qRT-PCR analysis of GFP expression in the infiltrated leaf areas. Total RNAs were extracted from leaves of N. benthamiana infiltrated with the pCanG-HA-GFP or pCanG-ABI4-GFP combined with Pro-CYP707A1-LUC (A) or Pro-CYP707A2-LUC (B) constructs. Tobacco Actin was employed as the internal control in the qRT-PCR analysis. The experiments performed three biological repeats and obtained the similar trend. (C) and (D) using the PCR-DNA amount of promoters to represent the equal plasmid DNA in agro-infiltration were applied between parallel experiments in Figure 5. (TIF) [file pgen.1003577.s005.tif]

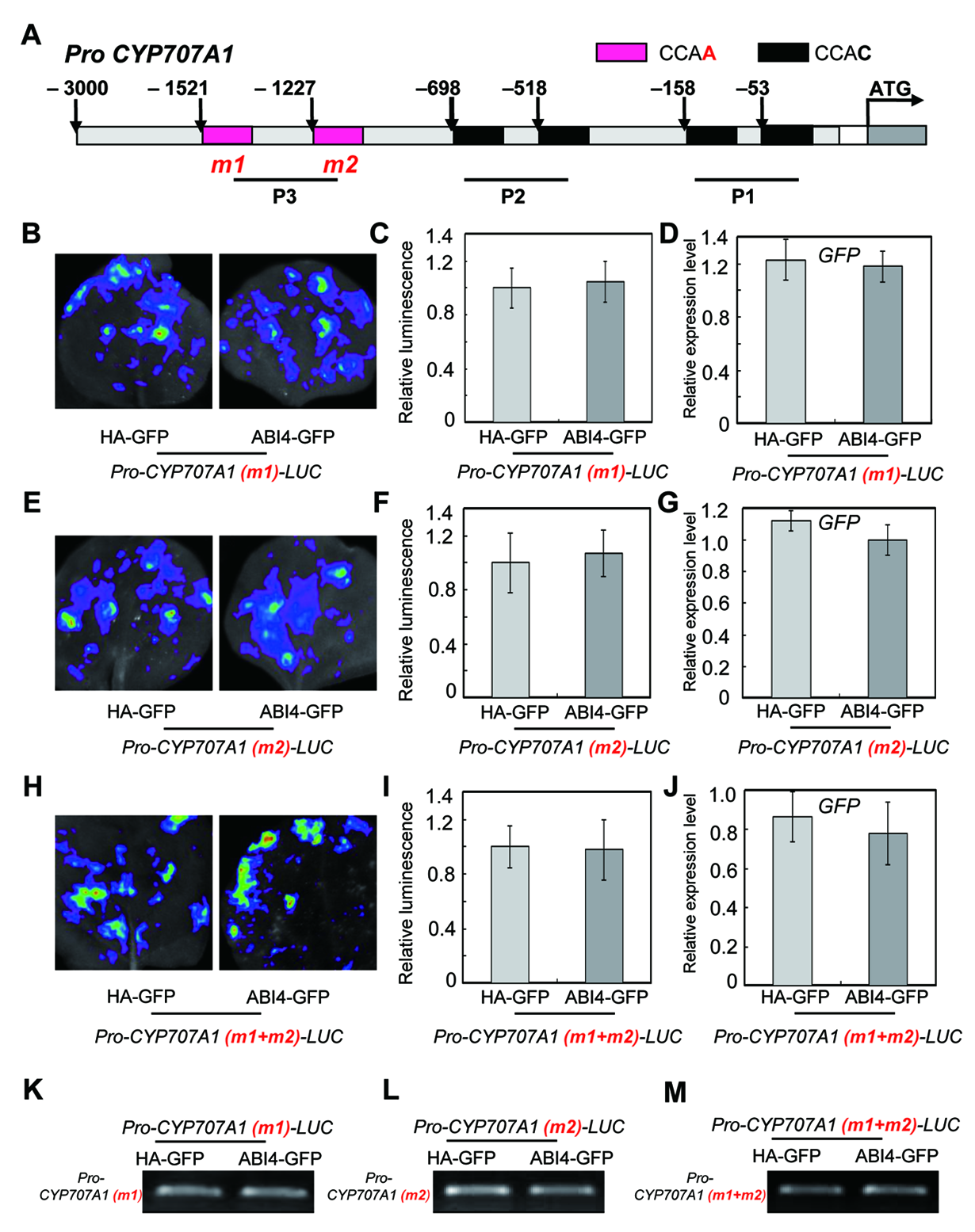

Supplement: Figure S6 — ABI4 could not inhibit CYP707A1 transcription when the CCAC cis-elements were mutated. (A) Mutated scheme of promoter of CYP707A1. The two CCAC motifs in P3 region were changed to CCAA. (B)–(M) Different mutated forms of CYP707A1 promoter were analyzed. Representative images of N. benthamiana leaves are shown in (B), (E) and (H). The corresponding quantitative analyses of luminescence intensity are shown in (C), (F) and (I). (D), (G) and (J) represented the GFP expression in the infiltrated tobacco leaves for the different combinations. Total RNAs were extracted from leaves of N. benthamiana leaves. The experiments performed three biological repeats and obtained the similar trend. Tobacco Actin was employed as the internal control in qRT-PCR analysis. (K) to (M) Using the PCR-DNA amount of promoters to represent the equal plasmid DNA in agro-infiltration were applied between parallel experiments. (B)–(D) For Pro-CYP707A1 (m1)-LUC. (E)–(G) For Pro-CYP707A1 (m2)-LUC. (H)–(J) For Pro-CYP707A1 (m1+m2)-LUC. (TIF) [file pgen.1003577.s006.tif]

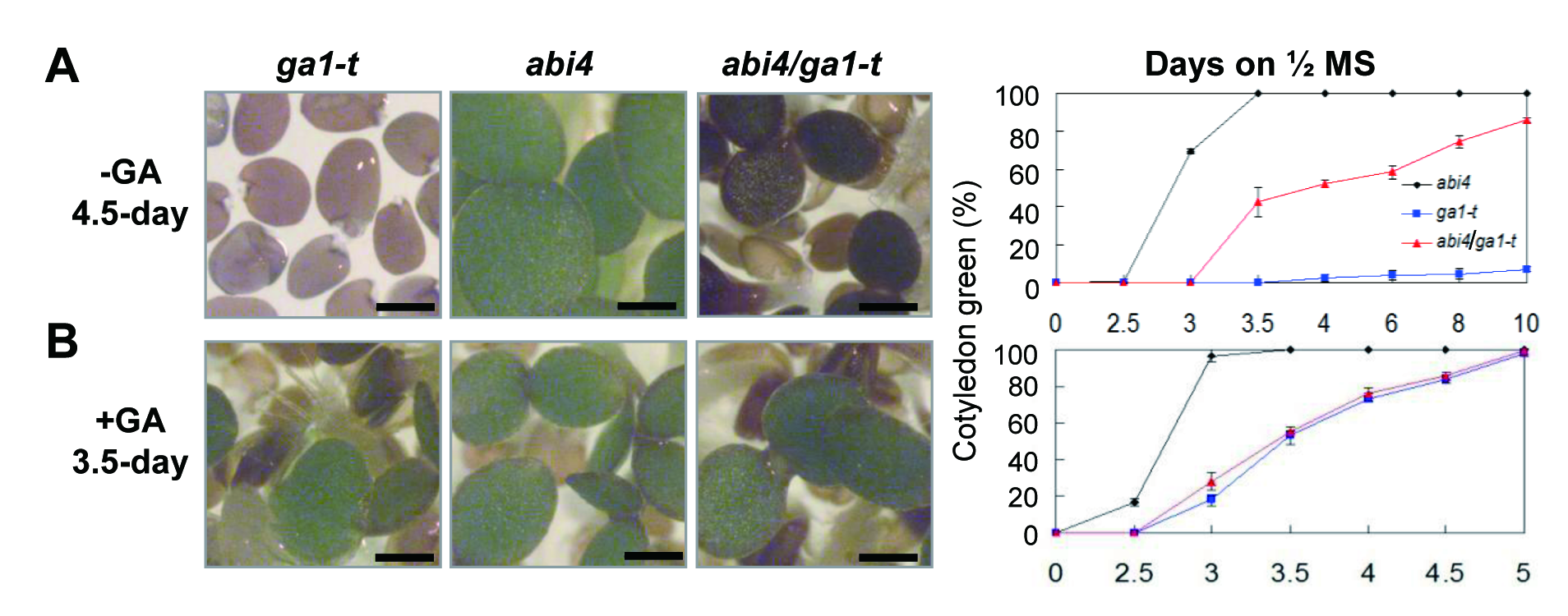

Supplement: Figure S7 — Cotyledon greening rates of abi4, ga1-t and abi4/ga1-t with or without exogenous GA treatment. Quantitative analysis results were shown in the right panels (n≥45). One representative image (time points indicated in figures) per genotype is shown (left panels). Bar = 0.25 mm. Percentages are the average of three repeats ± standard error. (A) Cotyledon greening of abi4, ga1-t and abi4/ga1-t mutants in the absence of exogenous GA treatment. (B) Cotyledon greening of abi4, ga1-t and abi4/ga1-t mutants in the presence of exogenous GA treatment. (TIF) [file pgen.1003577.s007.tif]

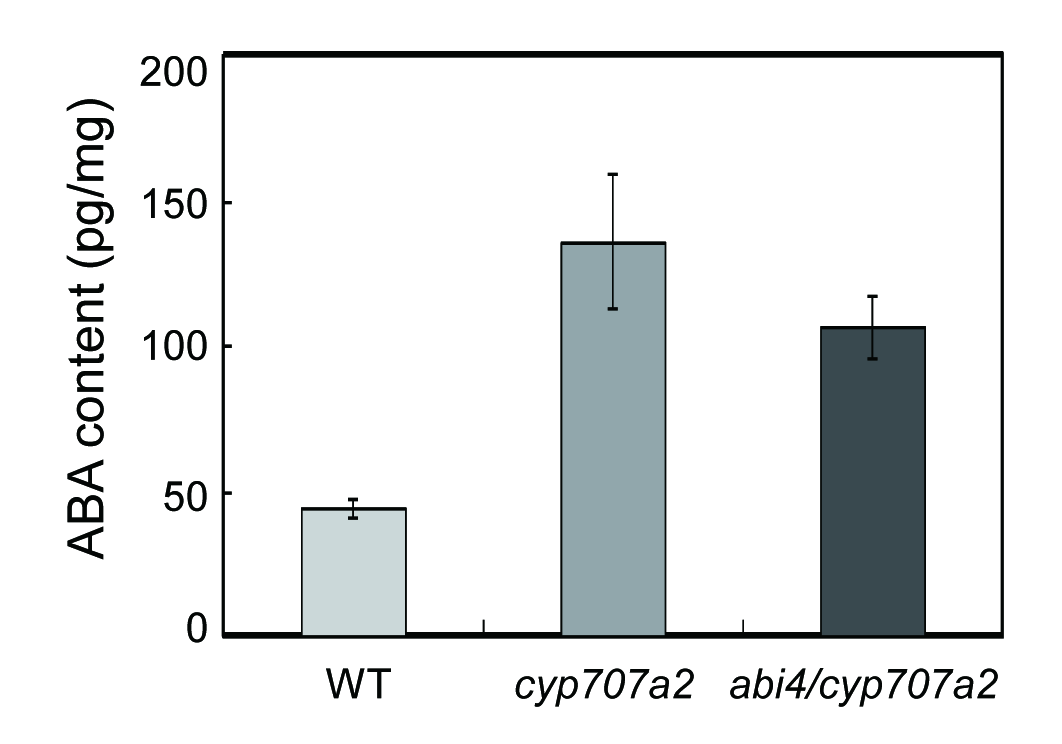

Supplement: Figure S8 — Endogenous ABA measurements in different genotypes. Endogenous ABA levels in WT, cyp707a1 and abi4/cyp707a1 seeds were quantified. Two-week stored seeds were used for analysis. (TIF) [file pgen.1003577.s008.tif]
