# Supplementary material for: ABI4 Regulates Primary Seed Dormancy by Regulating the Biogenesis of Abscisic Acid and Gibberellins in Arabidopsis
Source: PLoS Genet. 2013 Jun 20;9(6):e1003577. doi: 10.1371/journal.pgen.1003577 (PMC3688486; doi:10.1371/journal.pgen.1003577)
Supplement: Table S1 — Primer sequences used in this study. (DOC) [file pgen.1003577.s009.doc]

**Table S1 Primer sequences used in this study.**

| **For the several transgenic constructs** | |
| --- | --- |
| pCanG-ABI4-GFP-Fw | tctagaATGGACCCTTTAGCTTCCCA |
| pCanG-ABI4-GFP-Rev | tctaga ATAGAATTCCCCCAAGATGG |
| ProCYP707A1-LUC-Fw | caccGAGGGGGGAGAGGAAGAAAT |
| ProCYP707A1-LUC-Rev | ATACCTGTCATAAAACATCATCAAA |
| ProCYP707A2-LUC-Fw | caccAGATAGATGTTTTACAAAACTACATT |
| ProCYP707A2-LUC-Rev | TATTCCGAGTGATTTAAGTGAATTATTG |
| ProCYP707A1-LUC-m1-Fw | GAGGATTCGGT**CCAA**AATTCTCC |
| ProCYP707A1-LUC-m1-Rev | GGAGAATT**TTGG**ACCGAATCCTC |
| ProCYP707A1-LUC-m2-Fw | CATCTAGAATG**CCAA**ACATCTTC |
| ProCYP707A1-LUC-m2-Rev | GAAGATGT**TTGG**CATTCTAGATG |
|  |  |
| **For the qRT-PCR assay** | |
| qRT-GA20OX1 Fw | TATCGAACGATAGATACAAGAGC |
| qRT-GA20OX1 Rev | TATCTTCTTGATGTGATGCTGTC |
| qRT-GA20OX2 Fw | GAGCAGTTTGGGAAGGTGTATC |
| qRT-GA20OX2 Rev | TAACGTGAGATCTGGTGTTTGG |
| qRT-KAO1 Fw | GGAATTCTTATCGCAGGTTGTC |
| qRT-KAO1 Rev | AGCAGGATCAAATTTTCTTGGA |
| qRT-GA3 Fw | GCATAAACGTAGACTCGCAGTG |
| qRT-GA3 Rev | AGACGATCTTGGACACTTGGAT |
| qRT-GA3OX1 Fw | GGTCTAGCAGCTCATACCGACT |
| qRT-GA3OX1 Rev | CAACACGCTTTTAAACAATCCA |
| qRT-GA20ox3 Fw | TCGTGGACAACAAATGGCA |
| qRT-GA20ox3 Rev | TGAAGGTGTCGCCTATGTTCAC |
| qRT-KAO2 Fw | CAAAAGGCTGGAAGGTTCTGACTTG |
| qRT-KAO2 Rev | TCCCAAACCAAAAGGAAGGAATGTG |
| qRT-GA2ox8 Fw | TCGGAATCAGAGGCATTAGCATATATGTTGGCAGAGG |
| qRT-GA2ox8 Rev | GGTTTGGGACAAGGTGGATATCGGTTCATCCTTAGATAAC |
| qRT-RGL3-Fw | ATGGATACAGAGTGGAGGAGAACG |
| qRT-RGL3-Rev | GATGCAGCGATTAGAGGTTTCG |
| qRT-NCED2-Fw | TGCAGATCGACGTAACGGAATT |
| qRT-NCED2-Rev | GAAGATGTTTAGCCGGAGAGGAT |
| qRT-NCED3-Fw | AGGTCGCAAGATTCGGGATT |
| qRT-NCED3-Rev | GCGGATTTCAGACAGGACACTC |
| qrt-CYP707A1-fw | TCATCTCACCACCAAGTA |
| qrt-CYP707A1-rev | AAGGCAATTCTGTCATTCTA |
| qRT-CYP707A2 -Fw | ATCCATCACTCCTCCGAATTCTTCC |
| qRT-CYP707A2 -Rev | TCCATTTCCGAATGGCATGTACG |
| qRT-CYP707A3-Fw | CATGCCTTTTGGTAGTGGGATTCAT |
| qRT-CYP707A3 -Rev | CGGCCCATACTGAATTCCATCG |
| qRT-ABI4 Fw | GTCCAGATGGGACAATTCCAACACC |
| qRT-ABI4 Rev | CCCTAACGCCACCTCATGATGAAAC |
| qRT-ABI5 Fw | AAGCCACCGGTTTTTAGACACACAG |
| qRT-ABI5 Rev | CACCTCCTCCATTATGTCTCGCTTG |
| qRT-18S-Fw | GTTGATCCTGCCAGTAGT |
| qRT-18S-Rev | ATCCGAGTAGTAGTTACCATC |
| Nt-Actin-RT-Fw | CATCAGGAAGGACTTGTACGG |
| Nt-Actin-RT-Rev | GATGGACCTGACTCGTCATAC |
| qRT-GFP-Fw | ATGGGTAAAGGAGAAGAACT |
| qRT-GFP-Rev | AGTAAGAGTAGTGACAAGTG |
|  |  |
| **For ChIP-qPCR assay** | |
| qrt-ChIP-ABI5-1-Fw | CCTGTCTAAGTTAGCATTCCATTG |
| qrt-ChIP-ABI5-1-Rev | GGTTCTCCTCCTTCACATAGTT |
| ChIP-CYP707A1-P1-Fw | CCTTCACATCTCCCACTTGT |
| ChIP-CYP707A1-P1-Rev | GTGAGAAACAAGGCGGAGAT |
| ChIP-CYP707A1-P2-Fw | TCCCACTCTTTTATTCACTC |
| ChIP-CYP707A1-P2-Rev | AAATGTGGGGTAAAGTCTAC |
| ChIP-CYP707A1-P3-Fw | AAAGATGATGAGGATTCGGT |
| ChIP-CYP707A1-P3-Rev | GTAAAATGAAGATGTGTGGC |
| ChIP-CYP707A2-P4-Fw | CGTGGATTTCTAGGGATGTC |
| ChIP-CYP707A2-P4-Rev | CTTCATCATATCTTGGACCT |
| ChIP-CYP707A2-P5-Fw | AAGTGTAGTGTGGGGTTAGC |
| ChIP-CYP707A2-P5-Rev | CGCAGTACTATTTATGTGGT |
| ChIP-CYP707A3-P6-Fw | TTCCTCATCCTTCTCTCTCC |
| ChIP-CYP707A3-P6-Rev | TGGTTTTTGTGTTGGAGATG |
| ChIP-CYP707A3-P7-Fw | GGTTTTGATTTTTGTTGGGG |
| ChIP-CYP707A3-P7-Rev | TAAACGGTAATGGGAAAACC |
| ChIP-CYP707A3-P8-Fw | GGCCACAACCTTGATAAGAC |
| ChIP-CYP707A3-P8-Rev | GAACTATAGTACGTAAAGTTAATGTGG |
| TUB4-Fw | CGAGAGGATCACAGCAATACAG |
| TUB4-Rev | GGATCCATTCCACAAAGTAGGA |
|  |  |
| **For mutants verification** | |
| SALK_080095-LP | TGAATGCCTTGGAGTGTTTTC |
| SALK_080095-RP | GTGTTGGAATTGTCCCATCTG |
| SALK_023192-RP | TTGGACATGGGAACATCTAGC |
| SALK_023192-LP | CAATCTTCTTTTGAAGAGTTTCAATC |
| SALK_069127 -LP | CATGAACGTATTGGGTTTTGG |
| SALK_069127 -RP | TCCTGATATTGAATCCATCGC |
| SALK_083966C-LP | AATCCCAAATATGCCTTAGGC |
| SALK_083966C-RP | TATGTGGGGACTTTGATGGAC |
| SALK_026545C -LP | CGTAAGAATCAAACTGTTACATCAG |
| SALK_026545C -RP | CCGAAACCGAATACATTCATG |
| LBb1.3 | ATTTTGCCGATTTCGGAAC |
| dCAPs-abi4-1-Fw （CS8104） | GCCACCGTAGGAGGAGGATC |
| dCAPs-abi4-1-Rev（CS8104） | TGTTGGAATTGTCCCATCTGGA |
